# Advancing the frontiers of phytotherapy: a comprehensive review of botanical interventions targeting mitochondrial quality control to ameliorate endometriosis

**DOI:** 10.3389/fcell.2026.1813085

**Published:** 2026-04-29

**Authors:** Xi Ye, Xuanxuan Hong, Yingping Liu, Zhen Wang, Xiangfeng Zhang, Yingao Zheng, Le Yu, Liehong Wang

**Affiliations:** 1 Department of Obstetrics and Gynecology, Qinghai University Affiliated Clinical Medical College, Xining, China; 2 Department of Obstetrics and Gynecology, Hefei Maternal and Child Healthcare Hospital, Hefei, China; 3 Department of Obstetrics and Gynecology, Anhui Medical University, Hefei, China; 4 Department of Obstetrics and Gynecology, Hefei Eighth People’s Hospital, Hefei, China; 5 Department of Obstetrics and Gynecology, Anhui Provincial Second People’s Hospital, Hefei, China; 6 Department of Obstetrics and Gynecology, Qinghai Red Cross Hospital, Xining, China

**Keywords:** endometriosis, mitochondrial quality control system, phytomedicine, plant active ingredients, regulatory mechanism

## Abstract

Endometriosis (EMs) is a gynecological disorder affecting women of reproductive age. It is characterized by the ectopic implantation and infiltration of endometrial-like tissue and is associated with significant effects on fertility, pelvic function, and overall wellbeing. Clinically, it presents with progressive secondary dysmenorrhea, chronic pelvic pain, and infertility, leading to a substantial reduction in quality of life. Despite decades of research, the pathogenesis of EMs remains complex and is driven by interacting hormonal, immunological, inflammatory, and metabolic factors. Current treatment approaches, including hormonal suppression and surgical excision, provide only temporary relief and are associated with systemic side effects, functional limitations, and high recurrence rates. These limitations highlight the need for safer and more effective therapeutic strategies. Mitochondria play a central role in the development of EMs. Mitochondrial function is regulated by the mitochondrial quality control (MQC) system, which consists of several interconnected processes, including redox homeostasis, mitochondrial fission and fusion, mitophagy, biogenesis, and calcium signaling. Increasing evidence suggests that disruption of MQC is not merely a secondary effect but a key contributor to EMs pathogenesis. MQC dysfunction enables ectopic endometrial cells to evade apoptosis, promotes invasive proliferation, supports oxidative stress adaptation, and facilitates survival in unfavorable microenvironments. Plant-derived compounds have gained attention as potential modulators of MQC. These compounds exhibit multi-target effects, favorable safety profiles, and a wide range of bioactive structures. Experimental studies indicate that botanical agents, including flavonoids and terpenoids, can regulate multiple components of the MQC system. They reduce mitochondrial reactive oxygen species, modulate DRP1/OPA1-mediated mitochondrial dynamics, enhance PINK1-Parkin–dependent mitophagy, promote PGC-1α–mediated mitochondrial biogenesis, and maintain calcium homeostasis. Through these mechanisms, phytochemicals improve mitochondrial function, inhibit abnormal cell survival signaling, induce apoptosis in ectopic lesions, and contribute to the restoration of endometrial homeostasis.

## Graphical Abstract

**Figure:**
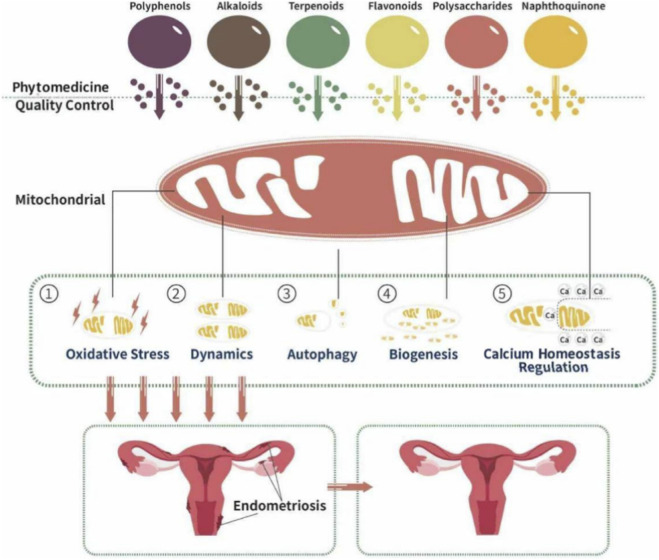

## 1 Introduction

Endometriosis (EMs) is a systemic gynecological disorder characterized by the aberrant presence and progressive establishment of endometrial-like glands and stroma outside the uterine cavity (Wilder et al., 2026; Charifson et al., 2026; Emadi et al., 2026). These ectopic implants do not remain passive. They actively infiltrate pelvic structures, including the ovaries, uterosacral ligaments, peritoneum, and, in some cases, distant extrapelvic sites. Although histologically benign, they exhibit biologically aggressive behavior. With an estimated prevalence of 5%–10% among women of reproductive age (Di et al., 2026), EMs is substantially more common in specific clinical subpopulations, affecting 30%–40% of individuals with infertility and up to 40%–87% of those with chronic pelvic pain (Vallée and Ayoubi, 2026; Robin et al., 2026). Although non-malignant in origin, EMs shares several features with malignant disease, including local tissue invasion, immune evasion, and frequent recurrence after treatment. This persistent recurrence, together with its strong hormonal dependence, particularly during the reproductive years between ages 25 and 45, indicates that EMs is not only a reproductive disorder but also a chronic inflammatory and metabolic condition. Genetic susceptibility adds further complexity, as first-degree relatives of affected individuals have a 3- to 9-fold increased risk (Metzler et al., 2024), suggesting a heritable contribution involving epigenetic and immunogenetic mechanisms. Current management is guided by four major therapeutic goals: lesion suppression, symptom relief, fertility preservation, and recurrence prevention. Clinical strategies include expectant management, hormonal therapy, and surgical excision, each with important limitations. Prolonged hormonal therapy can relieve symptoms, but it may also lead to adverse effects such as skeletal demineralization, metabolic disturbance, and menstrual disruption. Laparoscopic surgery provides visible lesion removal, but microscopic residual disease often remains undetected and unresected, creating the basis for recurrence. As a result, current approaches remain largely palliative rather than curative, highlighting the need for integrative and mechanism-based therapeutic strategies targeting cellular resilience, immune regulation, and mitochondrial function (Kolben et al., 2026; Pinto et al., 2026).

Mitochondria, as dynamic double-membraned organelles and central regulators of cellular fate, perform functions far beyond ATP production. They are essential for energy metabolism, redox signaling, calcium buffering, and activation of the intrinsic apoptotic pathway. When mitochondrial integrity is disrupted, a cascade of dysfunction follows, including bioenergetic failure, excessive oxidative stress, impaired calcium homeostasis, and escape from programmed cell death. These changes together promote pathological phenotypes such as uncontrolled proliferation, tissue invasion, and therapeutic resistance across a wide range of chronic diseases (Wang G. et al., 2025). At the core of mitochondrial resilience is the mitochondrial quality control (MQC) system, a coordinated and multilayered surveillance and renewal network composed of five major components: redox homeostasis, mitochondrial fission-fusion dynamics, mitophagic turnover, biogenic regeneration, and calcium signaling regulation. Together, these mechanisms enable the selective removal of irreversibly damaged mitochondria, repair of partially injured organelles, and generation of new functional mitochondria, thereby preserving metabolic activity, genomic stability, and cellular homeostasis (Dantas et al., 2026). In endometriosis (EMs), MQC is not simply altered but profoundly disrupted. Increasing evidence shows that ectopic endometrial cells contain fragmented mitochondria with reduced membrane potential, excessive ROS production, and impaired oxidative phosphorylation. Importantly, multiple components of the MQC system are affected, including reduced PGC-1α-driven biogenesis, excessive DRP1-mediated fission relative to OPA1/MFN2-dependent fusion, defective PINK1-Parkin-mediated mitophagy, and decreased mitochondrial unfolded protein response (UPRmt) activity. Together, these abnormalities contribute to a self-reinforcing cycle of mitochondrial dysfunction and cellular adaptation. This MQC failure is not merely a consequence of disease but an active contributor to it, enabling ectopic lesions to resist apoptosis, maintain invasive capacity, survive under hypoxic and inflammatory stress, and persist within hostile microenvironments, thereby promoting EMs progression and recurrence (Zhao et al., 2025). Plant medicine has emerged as a valuable source of bioactive compounds with therapeutic potential in this context. Constituents such as flavonoids, terpenoids, stilbenes, and polyphenolic glycosides exert pleiotropic and often synergistic effects, including reduction of ROS, modulation of signaling pathways involved in mitochondrial dynamics, enhancement of autophagic flux through AMPK/mTOR and SIRT1 signaling, promotion of mitochondrial biogenesis through NRF1/TFAM activation, and stabilization of calcium homeostasis. Owing to their multitarget actions and relatively low systemic toxicity, botanical agents offer a promising pharmacological profile for long-term management of complex disorders such as EMs (Geng et al., 2025). Mechanistic studies increasingly indicate that specific plant-derived compounds, particularly quercetin, curcumin, resveratrol, and ginsenoside Rb1, act as modulators of MQC by restoring mitochondrial membrane integrity, rebalancing fission-fusion dynamics, promoting the clearance of dysfunctional mitochondria, and reactivating apoptotic signaling in ectopic endometrial cells, thereby producing meaningful anti-EMs effects (Liu et al., 2025a). This review focuses on mitochondria as a central site of dysfunction in endometriosis and examines plant medicine as a potential therapeutic strategy. We summarize current evidence on how botanical interventions regulate MQC structure and function, identify the molecular targets and pathway-specific effects of key phytochemicals, evaluate translational challenges related to dosing, delivery, and biomarker validation, and propose future directions for the development of mitochondria-targeted therapies for EMs.

## 2 Mitochondrial quality control

**FIGURE 1:**
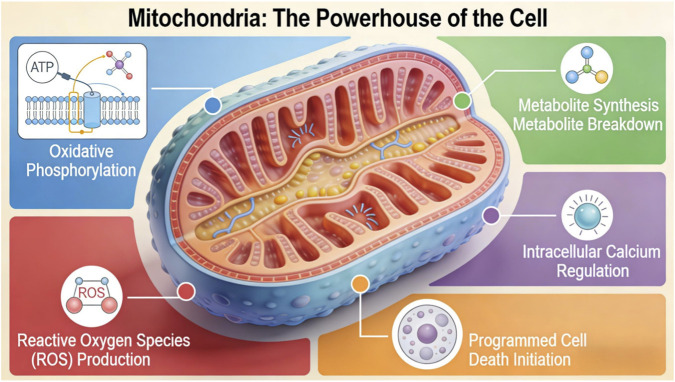
Mitochondria.

Mitochondria are not merely biochemical organelles. They are dynamic regulators of essential cellular functions. Through oxidative phosphorylation, they synthesize adenosine triphosphate (ATP), which serves as the primary energy source for cellular processes. However, their role extends well beyond bioenergetics. Mitochondria function as central metabolic integrators by regulating the synthesis and catabolism of lipids, amino acids, and nucleotides. They also act as redox regulators by controlling reactive oxygen species (ROS), as calcium buffers by limiting cytosolic Ca^2+^ overload, as signaling platforms that influence pathways such as NF-κB, HIF-1α, and mTOR, and as key mediators of cellular fate through activation of the intrinsic apoptotic pathway (Yan et al., 2026) (see Figure 1).

The mitochondrial quality control (MQC) system functions as an endogenous, multilayered defense and renewal network that preserves and optimizes mitochondrial fitness. It maintains a dynamic balance among three major processes: (i) structural plasticity, which reshapes mitochondrial networks through coordinated fission and fusion to distribute components, isolate damage, and respond to metabolic demand; (ii) functional surveillance, which identifies damaged mitochondria through redox, membrane potential, and proteostatic signals and then either repairs them through chaperone-mediated refolding or removes them through selective mitophagy; and (iii) regenerative renewal, which replenishes the mitochondrial pool through PGC-1α-driven biogenesis and preserves genomic integrity through mtDNA maintenance mechanisms (Meichsner et al., 2026).

**FIGURE 2:**
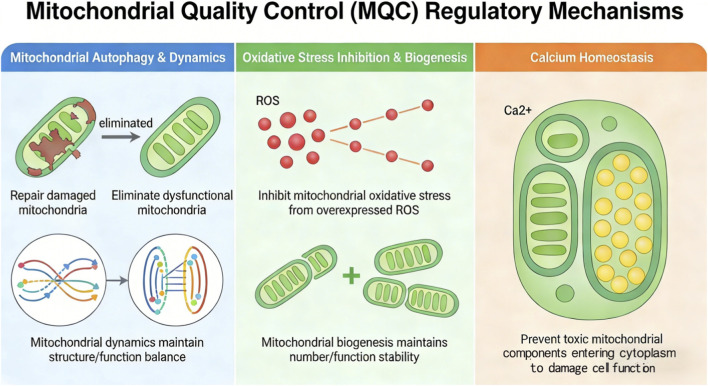
Mitochondrial quality control regulatory mechanisms.

Importantly, MQC functions as an integrated network rather than a set of isolated processes. Oxidative stress regulation, calcium homeostasis, mitochondrial dynamics, mitophagy, and biogenesis remain in continuous cross-talk to preserve bioenergetic stability, prevent cytotoxic leakage, and maintain cellular resilience. Among these components, mitochondrial calcium handling has a particularly important role. As a major intracellular Ca^2+^ buffer, mitochondria sequester transient calcium elevations, thereby preventing pathological cytosolic overload, while also using calcium as a metabolic signal to support ATP production. In addition, intact mitochondrial calcium homeostasis serves as an important protective barrier by confining potentially harmful mitochondrial contents, including cytochrome c, apoptosis-inducing factor (AIF), and mtDNA fragments, within the organelle and preventing their release into the cytosol, where they could trigger inflammation, necrosis, or abnormal apoptosis (see Figure 2).

Existing reviews have discussed endometriosis pathogenesis, epigenetic regulation, lesion migration and invasion, or natural plant metabolites (Marquardt et al., 2023; Mariadas et al., 2025; Wei et al., 2025; Yu Y. et al., 2025). However, these reviews do not systematically integrate mitochondrial quality control as a unifying framework linking disease mechanisms with phytotherapeutic intervention. The present review addresses this gap by organizing the evidence around oxidative stress, mitochondrial dynamics, mitophagy, biogenesis, and calcium homeostasis, and by mapping representative phytochemicals to these pathways (see Table 1).

**TABLE 1:** Comparison of recent review articles on endometriosis pathogenesis and therapeutic strategies, highlighting the distinct focus of the present review on mitochondrial quality control–targeted phytotherapy.

Review	Main focus	Main limitations	How current review differs
Marquardt et al. (2023)	Epigenetic dysregulation in endometriosis, including DNA methylation, histone modification, and chromatin regulation	Focuses on epigenetic mechanisms and epigenetically targeted therapeutics, but does not organize the field around mitochondrial quality control (MQC) or phytotherapy	This paper links MQC pathways to disease mechanisms and therapeutic opportunities from botanical compounds
Mariadas et al. (2025)	Broad molecular and cellular mechanisms of endometriosis, including genetic, epigenetic, immune, hormonal, and signaling pathways	Broad pathophysiology review; not specifically centered on MQC or on plant-derived mitochondrial regulators	This paper narrows the therapeutic framework to MQC-targeted phytotherapy
Wei et al. (2025)	Molecular mechanisms of migration and invasion in endometriosis and related pharmacologic inhibitors, including plant extracts and Chinese medicine	Focuses mainly on migration/invasion biology and related inhibitors rather than the full mitochondrial regulatory network	This review uses mitochondrial dysfunction/MQC as the organizing axis rather than lesion invasion alone
Yu Y. et al. (2025)	Systematic review of natural plant metabolites treating endometriosis by promoting apoptosis; systematic search identified 79 included studies	Strong phytochemical review, but its mechanistic lens is mainly apoptosis, not the broader MQC system	This review expands beyond apoptosis to include oxidative stress, mitochondrial dynamics, mitophagy, biogenesis, and calcium homeostasis
Present review	Phytotherapeutic modulation of mitochondrial quality control in endometriosis	—	Provides an integrated MQC-centered framework connecting phytochemicals to specific mitochondrial defects and future therapeutic strategies

## 3 Mitochondrial quality control system

### 3.1 Mitochondrial oxidative stress

Mitochondria function as major sources and regulators of reactive oxygen species (ROS). Within the mitochondrial matrix, controlled electron leakage during oxidative phosphorylation generates low levels of ROS. These ROS are not simply byproducts but serve as signaling molecules involved in cellular adaptation, proliferation, and immune regulation. This balance is maintained by an integrated antioxidant enzyme system, including mitochondrial superoxide dismutase (MnSOD), glutathione peroxidase (GPx), and thioredoxin reductase. These enzymes convert superoxide (O_2_•^-^) into hydrogen peroxide (H_2_O_2_), which acts as a secondary messenger in redox-sensitive signaling pathways such as NF-κB, MAPK, and HIF-1α (Yang et al., 2026; Kuşçu et al., 2026).

Mitochondrial oxidative stress occurs when this redox balance is disrupted. It arises when ROS production increases beyond physiological levels or when antioxidant defenses are impaired. Contributing factors include defects in the electron transport chain, environmental toxins, inflammatory cytokines, reduced MnSOD activity, glutathione depletion, and impaired thioredoxin function. Under these conditions, excess ROS initiates a cascade of mitochondrial dysfunction (Jain Tiffee et al., 2026).

Oxidative damage first affects the inner mitochondrial membrane, leading to pathological opening of the mitochondrial permeability transition pore (mPTP). This results in collapse of the electrochemical gradient and a rapid decline in mitochondrial membrane potential (MMP). As a consequence, the function of electron transport chain complexes I, III, and IV is impaired, and pro-apoptotic factors such as cytochrome c, SMAC/DIABLO, and apoptosis-inducing factor (AIF) are released into the cytosol.

**FIGURE 3:**
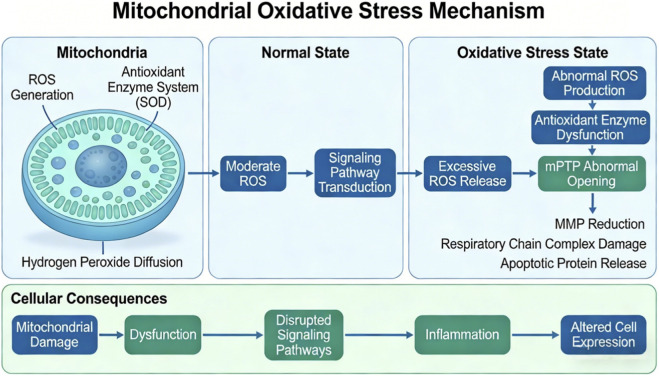
Mitochondrial oxidative stress mechanism.

These mitochondrial events have broader cellular consequences. Reduced ATP production compromises cellular energy supply. Release of mitochondrial DNA (mtDNA) activates NLRP3 inflammasomes and promotes chronic inflammation. Dysregulated ROS signaling alters transcription factors and kinase pathways. In addition, oxidative damage to lipids, proteins, and mtDNA further exacerbates mitochondrial dysfunction, creating a self-perpetuating cycle of cellular injury. As illustrated in Figure 3, these processes represent a coordinated pathogenic cascade rather than isolated mitochondrial damage.

### 3.2 Mitochondrial dynamics

Mitochondrial dynamics, defined by the coordinated processes of fission and fusion, plays an essential role in maintaining cellular homeostasis. It is not simply a structural adjustment but an active, energy-responsive system that continuously remodels the mitochondrial network to preserve genomic integrity, support oxidative phosphorylation, regulate calcium handling, maintain redox balance, and enable adaptive signaling. This dynamic regulation is fundamental for efficient energy metabolism, precise calcium control, and context-dependent intracellular communication, all of which are important for reproductive function, immune regulation, and cellular stress responses (Wang K. et al., 2026; Liang et al., 2026).

Mitochondrial fission, a controlled and GTP-dependent process, represents a key component of mitochondrial quality control. It enables the segregation of damaged or dysfunctional mitochondrial segments for selective autophagic removal, limits the propagation of ROS by isolating impaired regions, and ensures proper mitochondrial distribution during cell division. A central regulator of this process is dynamin-related protein 1 (Drp1), a cytosolic GTPase. Following post-translational modifications, including phosphorylation at Ser616 and ubiquitination, Drp1 is recruited to the outer mitochondrial membrane by adaptor proteins such as MFF, MiD49, and MiD51. There, it assembles into oligomeric structures that constrict the mitochondrion and mediate membrane division with high spatial and temporal precision (Wang K. et al., 2026).

In contrast, mitochondrial fusion serves as a complementary restorative mechanism. It allows the mixing of mitochondrial contents, thereby diluting accumulated damage, compensating for mtDNA defects, improving respiratory efficiency, and maintaining cristae structure required for ATP production. Fusion of the outer mitochondrial membrane is mediated by mitofusins 1 and 2 (MFN1/2), which interact through GTP-dependent dimerization. Fusion of the inner membrane is regulated by optic atrophy 1 (OPA1), a dynamin-like GTPase that facilitates membrane tethering, lipid mixing, and cristae organization, resulting in elongated and functionally integrated mitochondrial networks (Liang et al., 2026).

**FIGURE 4:**
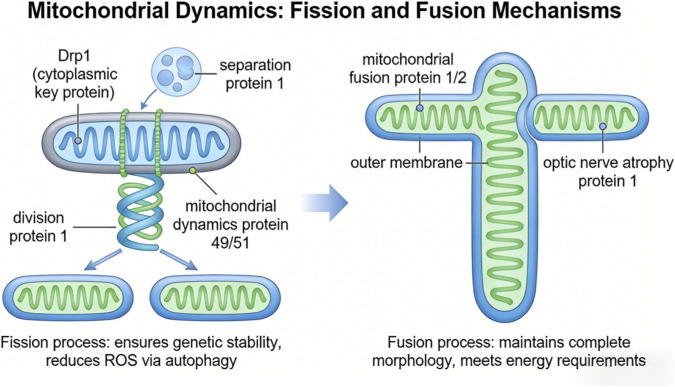
Mitochondrial dynamics.

Fission and fusion operate in a coordinated and interdependent manner. This balance forms an adaptive system that maintains mitochondrial integrity, supports cellular function, and contributes to long-term tissue homeostasis. As illustrated in Figure 4, mitochondrial dynamics represents a functional regulatory system rather than a purely structural phenomenon.

### 3.3 Mitochondrial autophagy

Mitochondrial autophagy, more accurately termed *mitophagy*, is a highly selective and evolutionarily conserved surveillance mechanism that identifies, tags, and eliminates aged, dysfunctional, or superfluous mitochondria while preserving healthy ones. It is not a passive degradative process but an active, signal-integrated quality control system that is essential for maintaining mitochondrial integrity, sustaining cellular bioenergetics, limiting oxidative stress, and preserving intracellular homeostasis, particularly in metabolically active and stress-sensitive tissues such as the endometrium and ovarian stroma (Dong and Zhang, 2024; Khan et al., 2026).

Two principal and functionally distinct mitophagic pathways regulate this process: the canonical PINK1-Parkin pathway and receptor-mediated, ubiquitin-independent pathways. Each pathway is activated in response to specific molecular signatures of mitochondrial damage.

In the PINK1-Parkin pathway, intact mitochondria continuously import and cleave PINK1 *via* the PARL protease, maintaining low steady-state levels. However, upon loss of mitochondrial membrane potential (ΔΨm), PINK1 import is disrupted, leading to its accumulation on the outer mitochondrial membrane (OMM). PINK1 then dimerizes and undergoes autophosphorylation, forming a signaling platform that phosphorylates both ubiquitin and the cytosolic E3 ubiquitin ligase Parkin. This results in the recruitment and activation of Parkin at the OMM, where it ubiquitinates multiple mitochondrial surface proteins. These polyubiquitinated proteins are subsequently recognized by autophagy adaptor proteins, including OPTN, NDP52, and TAX1BP1, which link ubiquitin chains to LC3-II on the expanding phagophore membrane. This coordinated process leads to the selective engulfment of damaged mitochondria into autophagosomes, which are then delivered to lysosomes for degradation (Dong and Zhang, 2024).

In contrast, receptor-mediated mitophagy operates through a ubiquitin-independent mechanism. Specific OMM-localized proteins, including FUNDC1, BNIP3, NIX/BNIP3L, and BCL2L13, function as LC3-interacting receptors (LIRs). Under conditions such as hypoxia, metabolic stress, or inflammation, these receptors undergo post-translational modifications, such as dephosphorylation or phosphorylation, that expose their LIR motifs. This enables direct interaction with LC3 or GABARAP proteins on nascent autophagosomes, allowing rapid and context-specific mitochondrial clearance without the requirement for upstream ubiquitination machinery. This pathway is particularly important in tissues that must rapidly adapt to fluctuations in oxygen availability or metabolic demand (Khan et al., 2026).

**FIGURE 5:**
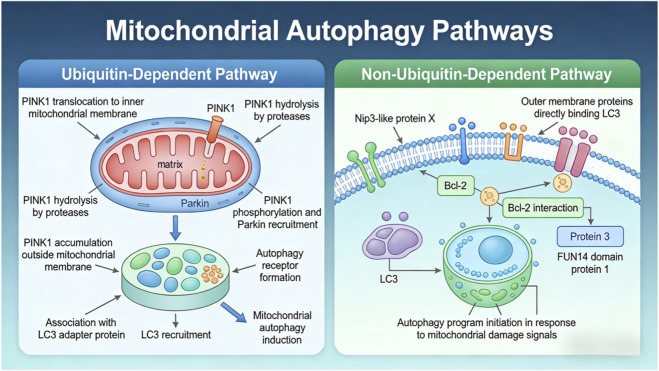
Mitochondrial autophagy pathways.

As illustrated in Figure 5, mitophagy represents a tightly regulated interface between mitochondrial quality control and overall cellular function. Disruption of this process leads to the accumulation of dysfunctional mitochondria, increased oxidative stress, activation of inflammatory pathways, and progressive cellular dysfunction, thereby contributing to the pathophysiology of disorders such as endometriosis.

### 3.4 Mitochondrial biogenesis

Mitochondrial biogenesis is the cell’s major regenerative program, involving a highly coordinated dialogue between the nucleus and mitochondria that directs the synthesis of new, functionally competent mitochondria to meet changing energetic, biosynthetic, and signaling demands. It represents more than a simple increase in mitochondrial number. Rather, it involves comprehensive renewal of the mitochondrial network, including increased mtDNA copy number, enhanced expression of respiratory chain subunits, expansion of cristae surface area, and improved oxidative phosphorylation capacity, while preserving genomic integrity and metabolic coordination. At the center of this transcriptional program is peroxisome proliferator-activated receptor gamma coactivator-1α (PGC-1α), which serves as a central regulator of mitochondrial biogenesis (Falk et al., 2026).

PGC-1α does not function as an independent transcription factor, but as a signal-responsive transcriptional coactivator whose activity is regulated by multiple converging pathways. It is strongly activated by AMP-activated protein kinase (AMPK), which phosphorylates PGC-1α at key residues and enhances its stability and nuclear translocation in response to energy depletion or metabolic stress. At the same time, sirtuin 1 (SIRT1) and SIRT3 deacetylate PGC-1α, increasing its interaction with transcription factors and enhancing its coactivator function under conditions of caloric restriction or redox stress (Falk et al., 2026).

Once activated, PGC-1α translocates to the nucleus and forms functional complexes with nuclear respiratory factors, primarily NRF1 and NRF2. These factors then promote the expression of mitochondrial transcription factor A (TFAM), a key mtDNA-packaging protein and transcriptional regulator. TFAM binds mtDNA with high affinity and promotes both replication and transcription of the mitochondrial genome, thereby supporting the synthesis of electron transport chain complexes, ribosomal RNAs, and tRNAs required for the generation of new, functional mitochondria (Shi H. et al., 2026).

**FIGURE 6:**
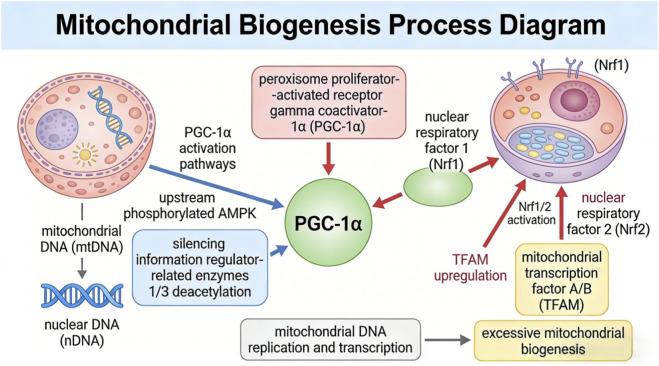
Mitochondrial biogenesis process diagram.

Importantly, this cascade is self-limited and context-dependent. Mitochondrial biogenesis increases only under physiologically relevant conditions, such as exercise adaptation, cold exposure, or tissue repair, and remains tightly regulated to prevent pathological overproliferation or energetic uncoupling. As illustrated in Figure 6, mitochondrial biogenesis is not uncontrolled expansion, but a regulated process of mitochondrial renewal that supports cellular resilience, endurance, and long-term metabolic health.

### 3.5 Mitochondrial calcium homeostasis regulation

Intracellular calcium (Ca^2+^) is not a passive ion but a dynamic second messenger whose spatiotemporal regulation has major effects on cellular survival and death. Although the endoplasmic reticulum (ER) serves as the main intracellular Ca^2+^ reservoir, mitochondria act as high-capacity and rapid-response Ca^2+^ buffers. They take up cytosolic Ca^2+^ through the mitochondrial calcium uniporter (MCU) complex located on the inner mitochondrial membrane and release it in a regulated manner through Na^+^/Ca^2+^ and H^+^/Ca^2+^ exchangers. This close ER-mitochondrial interaction, mediated by membrane contact sites known as mitochondria-associated membranes (MAMs), forms an important signaling interface that regulates bioenergetics, autophagy, apoptosis, and redox homeostasis (Guo et al., 2026).

Calcium homeostasis is therefore more than simple concentration balance. It is a tightly regulated system that is essential for mitochondrial function. Under physiological conditions, Ca^2+^ acts as an important metabolic activator. It stimulates key dehydrogenases in the tricarboxylic acid (TCA) cycle, including pyruvate dehydrogenase, isocitrate dehydrogenase, and α-ketoglutarate dehydrogenase, thereby increasing NADH production and enhancing electron flow through the respiratory chain. In parallel, Ca^2+^-dependent activation of F_1_F_0_-ATP synthase improves oxidative phosphorylation (OXPHOS) efficiency, while Ca^2+^-regulated kinases influence mitophagy initiation and mitochondrial dynamics, allowing energy production to adapt to cellular demand.

In contrast, pathological Ca^2+^ overload, induced by ER stress, excitotoxicity, or inflammatory signaling, exceeds the buffering capacity of mitochondria. This leads to opening of the mitochondrial permeability transition pore (mPTP), a non-selective channel whose prolonged activation collapses the proton motive force, dissipates mitochondrial membrane potential (MMP), uncouples OXPHOS, and induces cristae remodeling and mitochondrial swelling. These events lead to broader cellular consequences. Impaired ATP production compromises cellular integrity, release of pro-apoptotic factors activates both caspase-dependent and caspase-independent cell death pathways, and release of mitochondrial damage-associated molecular patterns (DAMPs) promotes sterile inflammation. In this way, a localized disturbance in ionic balance can progress to widespread cellular dysfunction.

**FIGURE 7:**
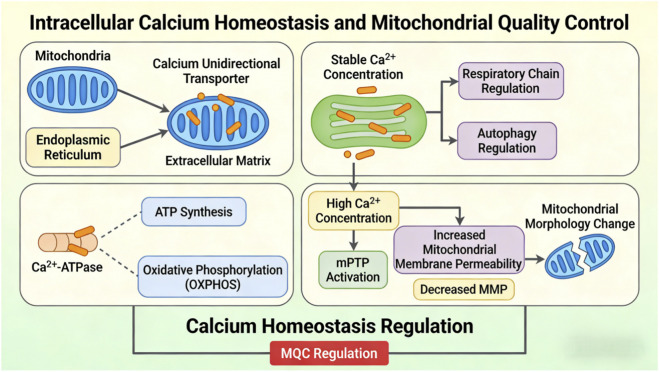
Intracellular calcium homeostasis and mitochondrial quality control.

Accordingly, calcium homeostasis is not simply one component of mitochondrial quality control (MQC), but a major integrative hub that coordinates interactions among oxidative stress responses, mitochondrial dynamics, mitophagy, and biogenesis to preserve metabolic stability and cellular longevity. As illustrated in Figure 7, mitochondrial calcium handling has a central role in MQC, and its dysregulation contributes directly to pathological dysfunction.

Mitochondrial metabolism is increasingly recognized as an important regulator of epigenetic processes. Several mitochondrial metabolites, including acetyl-CoA, α-ketoglutarate, and NAD^+^, serve as essential cofactors for enzymes involved in histone acetylation, histone demethylation, and DNA methylation. As a result, alterations in mitochondrial quality control may indirectly influence gene expression by modifying chromatin structure and epigenetic signaling pathways. In endometriosis, mitochondrial dysfunction and altered cellular metabolism may therefore contribute to abnormal epigenetic regulation of genes involved in inflammation, angiogenesis, and cell survival. Restoration of mitochondrial function by phytochemicals may thus have additional therapeutic implications through modulation of epigenetic pathways (Martínez-Reyes and Chandel, 2020; Santos, 2021).

## 4 The association between mitochondrial quality control systems and endometriosis

The mitochondrial quality control (MQC) system is a dynamic and integrated regulatory network in which redox homeostasis, mitochondrial fusion and fission, mitophagy, biogenesis, and calcium signaling operate in a coordinated manner to maintain mitochondrial integrity, metabolic function, and cellular resilience. Disruption of any single component of this system can affect multiple interconnected processes, leading to impaired energy production, increased oxidative stress, calcium imbalance, and resistance to apoptosis. In endometriosis (EMs), such MQC dysfunction is not incidental but plays a central role in disease progression, contributing to ectopic lesion survival, increased proliferation, persistent inflammation, and reduced treatment response. Therefore, understanding the molecular mechanisms underlying MQC disruption in EMs is important for identifying therapeutic targets. In this context, plant-derived compounds may exert beneficial effects by restoring redox balance, regulating mitochondrial dynamics, promoting clearance of damaged mitochondria, supporting biogenesis, and stabilizing calcium signaling, thereby providing a mechanistically guided, mitochondria-centered approach to treatment.

### 4.1 Mitochondrial oxidative stress and endometriosis

Mitochondrial reactive oxygen species (ROS) arise from two major and interconnected sources: electron leakage within the respiratory chain and dysregulated iron metabolism. Both converge on a common pathogenic outcome, namely, severe redox imbalance. Under physiological conditions, the electron transport chain (ETC) functions with high efficiency. However, even minor inefficiencies, particularly at Complex I and III, allow single electrons to escape and reduce molecular oxygen (O_2_) to superoxide (O_2_•^-^), the primary mitochondrial ROS. When this leakage increases due to ETC hyperactivity, structural defects, or substrate overload, the resulting ROS accumulation exceeds antioxidant capacity and initiates lipid peroxidation cascades that destabilize membranes, damage mtDNA, and impair oxidative phosphorylation (Tang et al., 2024). Notably, ectopic endometrial lesions show this pattern of respiratory hyperactivation, with increased levels of Complex I and cytochrome b, along with elevated matrix expression of ATP, SOD2, and ROS. These findings indicate not passive ROS accumulation, but a state of sustained oxidative stress, in which increased SOD2 likely reflects a compensatory response to rising superoxide levels (Felgueiras et al., 2022).

Iron dysregulation represents another critical contributor. Although iron is essential for heme synthesis and ETC function, its redox-active ferrous form (Fe^2+^) catalyzes Fenton reactions that convert H_2_O_2_ into highly reactive hydroxyl radicals (•OH). Because mitochondria are major sites of iron utilization and storage, they are particularly susceptible to iron overload. Voltage-dependent anion channels (VDACs), especially VDAC2 and VDAC3, are important regulators at the outer mitochondrial membrane, controlling metabolite exchange, calcium flux, and apoptosis-related signaling. Recent evidence shows that erastin, a canonical ferroptosis inducer, binds directly to VDAC2/3, disrupts mitochondrial iron homeostasis, and induces marked intramitochondrial iron accumulation. In murine EMs models, this results in substantial lesion regression through ferroptosis, an iron-dependent and lipid peroxidation-driven form of regulated cell death characterized by mitochondrial shrinkage, cristae collapse, and plasma membrane rupture. Importantly, erastin-treated lesions exhibit significantly increased iron concentrations, supporting VDAC-mediated iron dysregulation as a key mechanism (Le et al., 2025).

In addition to these processes, certain bioactive flavonoids, including quercetin, luteolin, and apigenin, can exert selective pro-oxidant effects in stressed cells. They induce pathological opening of the mitochondrial permeability transition pore (mPTP), collapse mitochondrial membrane potential (MMP), and increase ROS release beyond recoverable levels. This targeted oxidative response selectively induces apoptosis in ectopic endometrial stromal cells while sparing healthy eutopic tissue, showing that botanical agents may exploit the inherent redox vulnerability of EMs lesions for therapeutic benefit (Wulandari et al., 2024).

Taken together, these findings indicate that mitochondrial ROS in EMs is not a nonspecific byproduct, but a coordinated, iron-amplified, and respiration-driven signal of metabolic stress that contributes to lesion persistence, inflammation, and tissue remodeling. Therapeutic strategies that restore redox balance, normalize iron handling, and preserve mitochondrial integrity may therefore target a central mechanism in EMs pathogenesis.

### 4.2 Mitochondrial dynamics and endometriosis

The dynamic balance between mitochondrial fusion and fission is not simply a structural maintenance process, but a central regulator of mitochondrial integrity, metabolic flexibility, inter-mitochondrial complementation, and quality control. Through coordinated fusion, mitochondria exchange contents, thereby diluting damaged proteins, compensating for mtDNA mutations, and optimizing respiratory efficiency. Through regulated fission, they isolate dysfunctional segments for mitophagic removal, ensure proper inheritance during cell division, and generate metabolically adaptable units for stress responses. This balance is markedly disrupted in ovarian endometriosis (OEM), where ectopic stromal cells are exposed to a hostile microenvironment characterized by chronic inflammation, hypoxia, and persistent oxidative stress. Under these conditions, mitochondria shift into a pathological state characterized by excessive fission, fragmented networks, and impaired fusion capacity (Kobayashi et al., 2024).

At this regulatory interface, the Hippo pathway kinase Mst1 appears to play an important role. *In vitro* studies show that Mst1, transcriptionally activated by the nuclear receptor NR4A, translocates to the nucleus, where it interacts with p53 to promote Drp1 gene expression and Drp1 phosphorylation at Ser616, thereby inducing excessive mitochondrial fragmentation. This increase in fission, however, has a protective effect in this context because it sensitizes ectopic endometrial cells to apoptosis by promoting release and activation of the mitochondrial serine protease HtrA2/Omi, which cleaves and inactivates inhibitor-of-apoptosis proteins (IAPs), thereby weakening a major anti-apoptotic defense and reducing lesion invasion and migration (Huang Y. et al., 2022). Consistent with this, animal models of OEM show markedly reduced Mst1 levels in peritoneal fluid, along with lower Drp1 expression and decreased Ser616 phosphorylation, indicating that loss of this regulatory axis contributes to mitochondrial stabilization, resistance to apoptosis, and more aggressive lesion progression. In contrast, in patients with mild endometriosis-associated infertility, a different form of dysregulation is observed. RT-PCR analysis of oocytes shows widespread mtDNA depletion, which is a feature of impaired mitochondrial turnover, together with morphological evidence of excessive and unopposed fusion. This hyperfused phenotype suggests failure of fission-mediated quality surveillance, allowing genetically compromised mitochondria to persist and thereby impairing oocyte quality, fertilization capacity, and early embryonic development (Khashchenko et al., 2024). Thus, mitochondrial dynamics in EMs is not altered in a single direction, but is dysregulated in a bidirectional manner. Excessive fission supports invasive survival in established lesions, whereas insufficient fission compromises germline quality in reproductive settings. Restoration of this balance, not by forcing fragmentation or fusion, but by re-establishing context-appropriate mitochondrial plasticity, may represent an important strategy for precision intervention across the EMs spectrum.

### 4.3 Mitochondrial autophagy and endometriosis

Mitochondrial autophagy, or mitophagy, is a highly selective, damage-responsive clearance process that identifies, labels, and removes dysfunctional, depolarized, or aged mitochondria while preserving the healthy mitochondrial network. As a core component of mitochondrial quality control (MQC), mitophagy supports organelle renewal, limits ROS amplification, reduces inflammatory DAMP release, and maintains metabolic integrity. Its canonical regulatory pathway, the PINK1-Parkin axis, acts as a damage-sensing mechanism. When mitochondrial membrane potential (ΔΨm) is lost, PINK1 accumulates on the outer mitochondrial membrane (OMM), dimerizes, and phosphorylates both ubiquitin and the cytosolic E3 ligase Parkin. Activated Parkin then ubiquitinates OMM proteins, including mitofusins and VDACs, generating signals that recruit autophagy adaptor proteins such as OPTN, NDP52, and TAX1BP1. These adaptors then interact with LC3-II on phagophores to mediate selective engulfment. The resulting mitophagosome fuses with lysosomes, completing degradation and supporting mitochondrial renewal, a tightly regulated process required for cellular homeostasis (Gou et al., 2025).

In endometriosis (EMs), however, this surveillance system is disrupted. Impaired mitophagy allows damaged mitochondria to accumulate, resulting in leakage of pro-inflammatory mtDNA, amplification of oxidative stress, and suppression of intrinsic apoptosis. These changes reduce the capacity to recognize and eliminate ectopic endometrial cells and thereby facilitate their abnormal adhesion, survival, and implantation outside the uterine cavity (Vassilaki et al., 2025). Experimental evidence in human endometrial stromal cells (hESCs) further illustrates this disruption. Knockdown of CHCHD2 significantly reduces expression of the anti-apoptotic protein Bcl-2 while increasing the pro-apoptotic protein Bax, which translocates to the OMM, oligomerizes, and permeabilizes the membrane to initiate cytochrome c release and caspase activation (Ren et al., 2022). Importantly, this apoptotic resistance is reinforced at an upstream level. Mst1, through p53-mediated transcriptional repression, suppresses Parkin expression and thereby inhibits the PINK1-Parkin mitophagy pathway. The result is disruption of both mitochondrial clearance and apoptosis: reduced mitophagy allows dysfunctional organelles to persist, while impaired apoptosis enables ectopic cells to avoid elimination, creating a permissive environment in which lesions survive, invade, and progress to chronic, treatment-resistant disease (Deng et al., 2023). Thus, mitophagy in EMs is not simply reduced, but functionally disabled. Restoring this process would represent more than mitochondrial clearance alone. It could alter cellular fate by reactivating a mitochondrial checkpoint that separates healthy from damaged states and limits persistent lesion recurrence.

### 4.4 Mitochondrial biogenesis and endometriosis

Mitochondria are not only cellular energy producers but also dynamic, semi-autonomous signaling organelles whose function depends closely on the integrity and regulation of mitochondrial DNA (mtDNA). Unlike nuclear DNA, mtDNA lacks protective histones and has limited repair capacity, making it more susceptible to oxidative damage and mutation. However, its role extends beyond genetic coding. mtDNA encodes key subunits of the electron transport chain (ETC), regulates respiratory efficiency, influences reactive oxygen species (ROS) signaling, and, through its non-coding control region, provides a site for transcription factor binding and regulatory control. Mitochondrial biogenesis and function rely on coordinated interaction between mtDNA and nuclear DNA (nDNA). While nDNA encodes over 1,000 mitochondrial proteins, including those required for replication, transcription, and translation, mtDNA contributes essential components for oxidative phosphorylation (OXPHOS). This coordinated relationship supports cellular energy production, redox balance, and stress responses, making mtDNA variation an important factor in endometriosis (EMs) susceptibility and progression.

Population-based studies have identified mtDNA variants associated with increased EMs risk. The T16189C polymorphism, a homopolymeric C-tract variant located in the non-coding control region, has been linked to higher disease incidence, particularly when present with the A10398G substitution in the ND3 gene (Kobayashi and Imanaka, 2025). Additional evidence shows that differences in EMs prevalence across populations are associated with the distribution of specific mtDNA haplogroups and point mutations, suggesting that mtDNA variation contributes to mitochondrial dysfunction, altered bioenergetics, and increased oxidative stress in ectopic lesions (Peng et al., 2025).

Further support comes from the identification of mitochondrial estrogen receptor β (mtERβ), a splice variant of nuclear ERβ that is transported into mitochondria through a targeting sequence. In EMs patients, mtERβ is significantly overexpressed in ectopic lesions compared to eutopic endometrium and healthy controls (Luo et al., 2026). Within mitochondria, mtERβ binds to the mtDNA control region and enhances transcription of ETC genes, increasing mitochondrial respiration. This increased activity does not support normal homeostasis but instead contributes to disease processes. Elevated ATP production supports cytoskeletal changes required for invasion, increased ROS promotes inflammatory and angiogenic signaling, and enhanced metabolic activity allows cells to survive and proliferate in hypoxic and inflammatory environments. In this way, mtERβ links hormonal signaling with mitochondrial function and contributes to lesion development and persistence.

Together, these findings indicate that mtDNA is not simply a passive genetic element but an active and regulated component of disease biology. Its variants and regulators, including mtERβ, influence mitochondrial metabolism in ways that promote survival, adhesion, and expansion of ectopic endometrial tissue. Understanding this interaction between mitochondrial genetics and hormonal regulation is important for explaining EMs pathogenesis and for developing targeted mitochondrial therapies.

### 4.5 Mitochondrial calcium homeostasis regulation and endometriosis

Intracellular calcium (Ca^2+^) is not a static ion pool but a dynamic and spatially regulated signaling mediator whose precise fluxes influence processes ranging from mitochondrial bioenergetics to uterine contractility. Its homeostasis is maintained by three major components: the endoplasmic reticulum (ER) as the primary intracellular reservoir, the plasma membrane as the regulator of extracellular influx, and mitochondria as a high-capacity and rapid-response buffer that sequesters cytosolic Ca^2+^ surges through the mitochondrial calcium uniporter (MCU) to regulate metabolism, mitochondrial dynamics, and cell fate decisions. When mitochondrial integrity is impaired, as reflected by increased membrane permeability and collapse of the electrochemical gradient (ΔΨm), this buffering capacity is lost. The resulting Ca^2+^ overload initiates a damaging cascade. It hyperactivates dehydrogenases in the tricarboxylic acid (TCA) cycle, drives excessive electron transport and ROS generation, induces sustained opening of the mitochondrial permeability transition pore (mPTP), and activates Ca^2+^-dependent proteases and nucleases that disrupt cellular architecture, ultimately leading to intrinsic apoptosis (Hong et al., 2025).

Importantly, this Ca^2+^ dysregulation also extends beyond the mitochondria and affects tissue-level physiology. Excess cytosolic Ca^2+^ directly stimulates L-type voltage-gated calcium channels (VGCCs) in uterine smooth muscle, leading to pathological hypercontractility that is clinically expressed as severe dysmenorrhea, a hallmark symptom of endometriosis (EMs). In addition, chronic pelvic pain, characterized by lower abdominal distension, pressure, and deep-seated pain, is increasingly recognized as a consequence of Ca^2+^-mediated neuronal sensitization, inflammatory mediator release, and abnormal myometrial-stromal crosstalk. Thus, Ca^2+^ is not simply involved in EMs pathogenesis, but functions as a central mediator linking mitochondrial dysfunction, inflammatory signaling, smooth muscle abnormalities, and pain.

This central role is further supported by evidence showing that the L-type calcium channel Cav1.3 is markedly overexpressed in both human ectopic endometrial lesions and immortalized primary endometrial stromal cells (hEM15A). Upon exposure to prostaglandin E_2_ (PGE_2_), an inflammatory mediator elevated in the peritoneal fluid of EMs patients, hEM15A cells further increase Cav1.3 expression. This enhances Ca^2+^ influx, which subsequently activates ADP-ribosyltransferase (ARTD1/PARP1), leading to energy-consuming poly-ADP-ribosylation and caspase cleavage, not to induce apoptosis, but paradoxically to inhibit it. In this way, the PGE_2_-Cav1.3-PARP1 axis forms a self-reinforcing survival pathway in which inflammation promotes Ca^2+^ influx, and Ca^2+^ influx supports anti-apoptotic signaling, allowing ectopic cells to evade clearance and persist in hostile microenvironments (Hong et al., 2025).

Therefore, targeting Ca^2+^ signaling, particularly Cav1.3, may represent a dual therapeutic strategy by restoring mitochondrial resilience while also normalizing uterine contractility and pain-related signaling. In this context, Ca^2+^ becomes not only a symptom-associated factor but also a unifying mechanistic link in disease progression and a potential target for symptom relief and disease control.

## 5 Research status of phytomedicine in regulating mitochondrial quality control system for treating endometriosis

**FIGURE 8:**
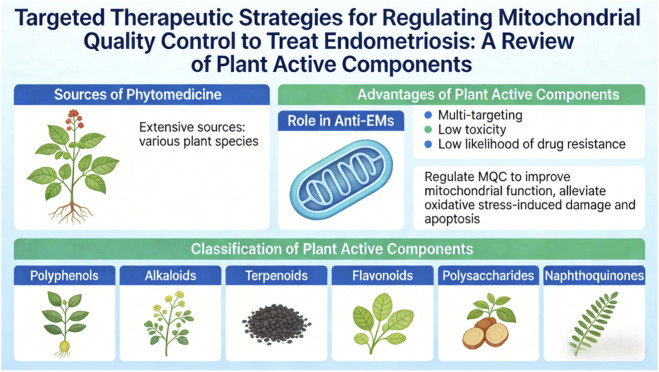
Targeted therapeutic strategies.

Phytomedicine, rooted in traditional healing systems and increasingly supported by modern pharmacognosy, represents a broad and chemically diverse source of therapeutic agents. Its active constituents, including polyphenols, alkaloids, terpenoids, flavonoids, polysaccharides, and naphthoquinones, have substantial molecular versatility. They act on multiple targets across interconnected signaling pathways, generally show favorable safety profiles with limited off-target toxicity, and may reduce the risk of resistance development, which remains a major challenge in chronic inflammatory disorders such as endometriosis (EMs). These properties have made phytomedicine an important area in anti-EMs drug development, offering not only symptomatic relief but also mitochondria-directed interventions that may restore bioenergetic function, reduce oxidative stress, rebalance fission-fusion dynamics, remove damaged organelles through mitophagy, and reactivate mitochondrial biogenesis (Wei et al., 2025; Yu Y. et al., 2025; Wang S. et al., 2025). Preclinical evidence indicates that diverse phytochemicals can modulate mitochondrial quality control (MQC) through defined molecular mechanisms. For example, epigallocatechin gallate (EGCG) chelates labile iron to suppress ferroptosis, berberine activates AMPK-PGC-1α signaling to enhance biogenesis and antioxidant defense, triptolide inhibits Drp1 phosphorylation to reduce excessive mitochondrial fission, curcumin upregulates Parkin and TFAM to promote mitophagy and mtDNA replication, and ginsenoside Rg3 stabilizes mitochondrial membrane potential (MMP) and inhibits mPTP opening. Together, these effects reduce oxidative damage, reverse apoptotic resistance, suppress NF-κB-driven inflammation, and limit lesion invasiveness (Ge et al., 2025). Therefore, the therapeutic potential of these compounds in EMs is supported by mechanistic and experimental evidence. Accordingly, this review adopts a structure-function framework, classifying plant-derived compounds not by botanical source but by chemical scaffold, including polyphenols, alkaloids, terpenoids, flavonoids, polysaccharides, and naphthoquinones, with each class linked to its major MQC target(s), validated molecular mechanism(s), and functional outcome(s) in EMs models. For each class, we discuss how specific structural features, such as catechol groups in polyphenols, isoquinoline cores in alkaloids, or α,β-unsaturated ketones in terpenoids, influence subcellular localization, receptor affinity, and enzymatic modulation, thereby contributing to selective restoration of mitochondrial homeostasis (see Table 2). As summarized in Figure 8, this scaffold-based framework positions phytomedicine not simply as a collection of natural compounds, but as a targeted approach to mitochondrial therapy in endometriosis.

**TABLE 2:** Representative phytochemicals that regulate mitochondrial quality control in endometriosis.

Compound	Class	Main MQC target(s)	Main mechanism(s) described in this review	Evidence stage in current manuscript
Curcumin	Polyphenol	Biogenesis, mitophagy, oxidative stress	Activates PGC-1α–NRF1–TFAM; upregulates Parkin/OPTN; preserves MMP; suppresses NF-κB and enhances Nrf2 antioxidant responses	*In vitro* + *in vivo*
EGCG	Polyphenol	Oxidative stress, dynamics, mitophagy	Activates Keap1–Nrf2–ARE; reduces ROS; stabilizes MMP; suppresses Drp1-mediated fission; supports PINK1/Parkin-dependent mitochondrial clearance	*In vitro* + *in vivo*
Resveratrol	Polyphenol	Biogenesis, oxidative stress, mitophagy	Activates AMPK–SIRT1–PGC-1α; improves antioxidant enzyme activity; stabilizes MMP; inhibits mPTP opening; promotes mitochondrial renewal	*In vitro* + *in vivo*
Tetrahydropalmatine (THP)	Alkaloid	Oxidative stress, apoptosis-related mitochondrial injury	Increases ROS in ectopic cells; induces ΔΨm collapse, mPTP opening, cytochrome c release, and caspase-dependent apoptosis	*In vitro* + *in vivo*
Ligustrazine	Alkaloid	Oxidative stress, mitochondrial membrane integrity, inflammation	Suppresses NLRP3 inflammasome; reduces oxidative stress; stabilizes ΔΨm; preserves ATP synthase activity; prevents mitochondrial swelling	*In vitro* + *in vivo*
Ginsenoside Rg3	Terpenoid saponin	Biogenesis, mitophagy, oxidative stress, membrane integrity	Activates PGC-1α–NRF1–TFAM; preserves ΔΨm; inhibits mPTP opening; promotes Parkin/LC3-II-related mitochondrial turnover; activates Nrf2 signaling	*In vitro* + *in vivo*
Tanshinone IIA	Terpenoid	Respiratory function, oxidative stress, mitochondrial structural repair	Restores ETC complex activity; preserves ΔΨm; reduces ROS leakage; improves mitochondrial membrane and cristae integrity	*In vitro* + *in vivo*
Quercetin	Flavonoid	Oxidative stress, biogenesis, mitophagy	Activates Keap1–Nrf2–ARE and AMPK/SIRT1; improves antioxidant defenses; stimulates PGC-1α–NRF1–TFAM; stabilizes ΔΨm; promotes Parkin-dependent mitophagy	*In vitro* + *in vivo*
Luteolin	Flavonoid	Dynamics, mitophagy, biogenesis, bioenergetics	Modulates Drp1/MFN1/2/OPA1 balance; promotes Parkin-mediated mitophagy; activates PGC-1α–NRF1–TFAM; enhances OXPHOS and ATP production	*In vitro* + *in vivo*
Spirulina polysaccharide	Polysaccharide	Oxidative stress, mitochondrial function	Upregulates SOD2; reduces oxidative injury; restores ΔΨm and respiratory function; also suppresses PI3K/Akt/mTOR-related lesion progression	*In vivo* for EMs; *in vitro* for mitochondrial mechanism
Laminarin	Polysaccharide	Stress signaling/angiogenesis with indirect mitochondrial relevance	Suppresses stress-related signaling and pathological angiogenesis in EMs models	*In vivo*
Shikonin	Naphthoquinone	Oxidative stress/apoptosis-related mitochondrial injury	Pro-apoptotic and redox-related anti-EMs effects discussed in the phytomedicine section	*In vitro* and/or *in vivo* if described in your full subsection

### 5.1 Polyphenolic plant active components

Polyphenolic compounds are a class of plant-derived compounds containing multiple phenolic hydroxyl groups. They are widely present in tea, grape seeds, and pomegranate and have strong antioxidant and anti-inflammatory effects. Their anti-endometriosis effects may be mediated through regulation of mitochondrial biogenesis and inhibition of oxidative stress.

Curcumin, the major bioactive constituent of turmeric (*Curcuma longa*) and other Zingiberaceae rhizomes, is a diarylheptanoid polyphenol with broad pharmacological activity (Jin et al., 2026). More than a conventional antioxidant, curcumin functions as an important regulator of mitochondrial quality control (MQC). It promotes biogenesis, strengthens antioxidant defenses, suppresses inflammatory signaling, stabilizes genomic integrity, and reactivates mitophagic turnover, thereby restoring key components of mitochondrial function that are disrupted in endometriosis (EMs). Preclinical evidence across multiple models supports these effects. *In vivo* studies show that curcumin significantly reduces ectopic lesion volume, inhibits fibrotic adhesion formation, and attenuates peritoneal inflammation (Wei et al., 2025). *In vitro*, it selectively suppresses proliferation of human ectopic endometrial stromal cells (hESCs) while strongly inducing intrinsic apoptosis through cytochrome c release and caspase-3 activation (Yu Y. et al., 2025). Mechanistically, curcumin exerts broad but specific mitochondria-centered effects. It activates the PGC-1α-NRF1-TFAM axis, the major transcriptional pathway governing mitochondrial biogenesis, thereby increasing mtDNA replication, enhancing oxidative phosphorylation (OXPHOS) capacity, and restoring ATP synthesis efficiency (Jannatifar et al., 2025). At the same time, it stabilizes mitochondrial DNA against oxidative damage, preserves mitochondrial membrane potential (MMP), and rebalances redox homeostasis by suppressing NF-κB-driven expression of pro-inflammatory cytokines such as TNF-α, IL-6, and IL-1β while increasing Nrf2-mediated antioxidant enzymes including SOD2, HO-1, and catalase (Jannatifar et al., 2025). Curcumin also functions as a potent enhancer of mitophagy. In animal models of EMs, it upregulates Parkin and OPTN expression, promotes LC3-II recruitment to damaged mitochondria, and accelerates autophagosome-lysosome fusion, thereby clearing dysfunctional organelles and preventing ROS accumulation, calcium dysregulation, and mPTP-driven collapse (Ding et al., 2023; Yönden et al., 2023). The overall effect is broad restoration of mitochondrial physiology, including normalized ROS flux, stabilized Ca^2+^ buffering, sustained ATP production, and preserved structural integrity, which together reduce lesion survival, impair invasive behavior, and restore cellular homeostasis. Thus, curcumin functions as a multi-targeted mitochondrial therapeutic in EMs.

Epigallocatechin gallate (EGCG), the most abundant and pharmacologically active catechin in *Camellia sinensis*, has important effects on mitochondrial function and cellular homeostasis in endometriosis (EMs). Its polyphenolic structure, containing a galloyl group and multiple phenolic hydroxyl groups, gives it strong redox-regulating capacity. This allows EGCG to scavenge ROS and reactive nitrogen species (RNS), chelate labile iron and copper, inhibit pro-oxidant enzymes such as xanthine oxidase, and activate the Keap1-Nrf2-ARE pathway to enhance endogenous antioxidant defenses including HO-1, NQO1, and SOD2. Beyond redox regulation, EGCG exerts multiple anti-EMs effects. It suppresses pathological angiogenesis by downregulating VEGF/VEGFR2 signaling and reducing microvessel density, induces G0/G1 cell cycle arrest and caspase-dependent apoptosis in ectopic endometrial stromal cells, inhibits TGF-β-Smad3-mediated collagen deposition to reduce fibrosis, and blocks MMP-2/9-mediated extracellular matrix degradation to impair lesion invasion and migration (Yönden et al., 2023). A major aspect of EGCG’s therapeutic effect is its mitochondria-centered mechanism. First, it directly improves mitochondrial function in EMs-like endometrial cells by restoring electron transport chain (ETC) efficiency, suppressing Drp1-mediated fission, enhancing MFN2 expression to promote fusion, and stabilizing mitochondrial membrane potential (MMP). Through these effects, EGCG reduces pathological ROS accumulation, reverses excessive proliferative activity, and restores intrinsic apoptotic competence (Hu et al., 2026). Second, it regulates mitochondrial quality control (MQC) by clearing excess cytosolic and mitochondrial ROS, protecting cardiolipin and ETC complexes from oxidative injury, preserving outer and inner membrane integrity, and selectively modulating mitophagy through PINK1-Parkin-dependent clearance of depolarized organelles, thereby maintaining a healthy and functional mitochondrial pool (Hu et al., 2026). In addition, EGCG suppresses the oncogenic STAT3 pathway by blocking IL-6-induced phosphorylation, nuclear translocation, and transcription of survival genes such as *Bcl-2*, *Mcl-1*, and *cyclin D1*, thereby complementing its mitochondrial actions at both organelle and nuclear levels (Yönden et al., 2023). To address its pharmacokinetic limitations, including low oral bioavailability and rapid metabolism, advanced delivery systems such as lipid-polymer hybrid nanoparticles, phospholipid complexes, and nanoemulsions have been developed. These approaches significantly improve cellular uptake, mitochondrial targeting, and sustained intracellular release, supporting the development of EGCG as a clinically useful mitochondria-directed therapeutic (Situmorang et al., 2026).

Resveratrol, a stilbenoid polyphenol widely found in the skin of red grapes, Japanese knotweed (*Polygonum cuspidatum*), and peanuts, is more than a dietary antioxidant. It is a potent sirtuin activator and an important regulator of mitochondrial function whose anti-endometriosis (EMs) effects are mediated through coordinated suppression of inflammation, restoration of redox balance, and recovery of bioenergetic function. Its therapeutic effects have been demonstrated across multiple experimental models. *In vitro*, resveratrol exerts strong immunomodulatory effects by significantly downregulating mRNA and protein expression of major chemokines and cytokines in ectopic endometrial stromal cells, including monocyte chemoattractant protein-1 (MCP-1), interleukin-6 (IL-6), interleukin-8 (IL-8), and RANTES (CCL5). By suppressing this inflammatory chemokine network, resveratrol reduces leukocyte recruitment, limits macrophage polarization toward the pro-inflammatory M1 phenotype, and restores immune balance, thereby interrupting the chronic inflammatory environment that promotes EMs lesion establishment and progression (Sanlier et al., 2026). *In vivo*, resveratrol treatment in EMs rodent models produces a strong and dose-dependent increase in systemic and tissue antioxidant capacity. Serum and ectopic lesion tissues show marked increases in superoxide dismutase (SOD), glutathione peroxidase (GPx), and catalase activity, indicating not only direct antioxidant effects but also transcriptional enhancement of endogenous defense systems (Fan et al., 2025). These effects are closely linked to mitochondrial function, which is both a major source and target of oxidative stress. Resveratrol activates the AMPK-SIRT1-PGC-1α signaling axis. AMPK phosphorylation promotes SIRT1 deacetylase activity, which then deacetylates and activates PGC-1α, the major coactivator of mitochondrial biogenesis. This pathway promotes NRF1/TFAM-mediated mtDNA replication, increases expression of mitochondrial antioxidant enzymes such as SOD2, Trx2, and Prx3, enhances citrate synthase activity as a marker of mitochondrial mass and TCA cycle activity, and improves respiratory coupling efficiency. Together, these effects reverse mitochondrial dysfunction, reduce ROS leakage, and restore ATP synthesis efficiency (Gołąbek-et al., 2024; Karadogan et al., 2025; Patel et al., 2025). In addition, resveratrol stabilizes mitochondrial membrane potential (MMP), inhibits mPTP opening, and promotes mitophagic clearance of damaged mitochondria, thereby supporting both quantitative expansion and qualitative renewal of the mitochondrial network. Thus, resveratrol acts as a broad mitochondrial restorative agent that suppresses inflammatory signaling while rebuilding the energetic and redox systems required for cellular homeostasis and EMs resolution.

### 5.2 Alkaloid active components of plants

Alkaloids are a class of naturally occurring nitrogen-containing organic compounds found mainly in plants and characterized by alkaline properties. Their anti-endometriosis effects may be related to regulation of mitochondrial oxidative stress and autophagy-related processes.

Tetrahydropalmatine (THP), a bioactive aporphine alkaloid isolated from the roots of *Angelica sinensis* (Danggui) and other traditional medicinal herbs, is a pharmacologically versatile compound whose therapeutic profile extends beyond its classical use for analgesia and sedation. Modern pharmacological studies identify THP as a potent mitochondria-targeted anti-endometriosis (EMs) agent. It disrupts lesion viability through coordinated induction of oxidative stress-driven apoptosis, suppression of pro-survival signaling, inhibition of abnormal proliferation and migration, and restoration of redox homeostasis. *In vivo* studies in EMs rodent models show that THP administration significantly reduces ectopic lesion volume and weight while also normalizing systemic inflammation by lowering serum TNF-α levels and rebalancing epithelial-stromal crosstalk through modulation of epidermal growth factor (EGF) signaling, specifically by downregulating overexpressed EGF receptor (EGFR), phosphoinositide 3-kinase (PI3K), and serine/threonine kinase Akt (Wuputra et al., 2026). Mechanistically, THP exerts its anti-EMs effects through mitochondrial vulnerability. It selectively increases intracellular reactive oxygen species (ROS) in hyperproliferative ectopic endometrial cells, not as uncontrolled toxicity, but as a regulated redox trigger. This ROS increase causes mitochondrial membrane depolarization (ΔΨm collapse), opens the mitochondrial permeability transition pore (mPTP), releases cytochrome c into the cytosol, and activates caspase-9 and caspase-3, culminating in intrinsic apoptosis (Niğdelioğlu, 2025). In addition to apoptosis induction, THP also has multiple anti-metastatic effects. It suppresses glucose uptake by inhibiting GLUT1 translocation, reduces NF-κB nuclear translocation and transcriptional activity to lower inflammatory and anti-apoptotic gene expression, and modulates oncogenic long non-coding RNAs such as MALAT1 and HOTAIR that are involved in EMs cell invasion and colony formation (Dai et al., 2023). Importantly, THP does not indiscriminately damage mitochondria. Rather, it exploits the elevated basal ROS levels and metabolic inflexibility of ectopic cells, providing a therapeutic window that spares healthy tissue while selectively eliminating pathological cells. Thus, THP acts not only as an alkaloid with analgesic properties, but also as a targeted mitochondrial regulator that promotes lesion regression through integrated metabolic, signaling, and organelle-level mechanisms.

Ligustrazine, a bioactive pyrazine alkaloid isolated from the rhizomes of *Ligusticum chuanxiong* (Chuanxiong), a major herb in Traditional Chinese Medicine, is a multifunctional cytoprotective agent with anti-inflammatory, antioxidant, anti-fibrotic, anti-pyroptotic, and mitochondrial-stabilizing properties (Nigdelioglu Dolanbay et al., 2021). Beyond its classical circulatory effects, ligustrazine acts as a broad regulator of cellular integrity in endometriosis (EMs). It suppresses sterile inflammation, limits pathological remodeling, restores redox balance, preserves mitochondrial bioenergetics, and improves microvascular homeostasis. *In vivo*, chronic administration of ligustrazine to EMs murine models over 4 weeks produces marked therapeutic effects. Ectopic lesions show significant volumetric regression, serum levels of pro-inflammatory cytokines including IL-1β, IL-18, and TNF-α are significantly reduced, oxidative stress markers such as MDA and 8-OHdG decline, and the NLRP3 inflammasome is suppressed together with inhibition of caspase-1 activation, gasdermin D cleavage, and pyroptotic cell death (Yi et al., 2021). Additional validation in EMs rat models shows that ligustrazine improves not only lesion burden but also associated structural and functional abnormalities. It reduces tissue fibrosis by inhibiting TGF-β-driven epithelial-mesenchymal transition (EMT) and fibroblast-to-myofibroblast transdifferentiation (FMT), downregulates collagen I/III and α-SMA expression, and alleviates mechanical hyperalgesia, thereby addressing both lesion burden and pain in EMs (Yu et al., 2026). *In vitro*, ligustrazine exerts multi-target regulatory effects on ectopic endometrial stromal cells (hESCs). It arrests proliferation through G2/M phase blockade, suppresses MMP-2/9-mediated invasion, reverses EMT by upregulating E-cadherin and downregulating Snail, Slug, and vimentin, and induces intrinsic apoptosis through modulation of the Bax/Bcl-2 ratio and activation of caspase-3 (Xu et al., 2025). Its mitochondrial protective effects are both structural and functional. Ligustrazine stabilizes mitochondrial membrane potential (ΔΨm), preserves ATP synthase (Complex V) and Na^+^/K^+^-ATPase activity, maintains cristae architecture, and prevents mitochondrial swelling. These effects are achieved through four coordinated mechanisms: (i) direct scavenging of free radicals such as •OH and O_2_•^-^, (ii) inhibition of lipid peroxidation chain reactions through suppression of 4-HNE and MDA formation, (iii) antagonism of pathological Ca^2+^ overload, and (iv) enhancement of mitochondrial energy synthesis through improved substrate delivery and microcirculatory perfusion (Huang S. et al., 2022). In addition, ligustrazine markedly upregulates endogenous antioxidant defenses, particularly superoxide dismutase (SOD), thereby further strengthening cellular resistance to oxidative stress (Liu et al., 2025b). Thus, ligustrazine functions as an integrated mitochondrial regulator by protecting organelles from structural damage, sustaining energy production, limiting oxidative stress, and suppressing inflammatory cascades at their mitochondrial origin, thereby extending its traditional role into a mitochondria-centered therapeutic strategy for endometriosis.

### 5.3 Active components of Saponin-containing plants

Terpenoids are another important class of active compounds in plants. They can be classified into monoterpenes, sesquiterpenes, diterpenes, and related subclasses based on the number of carbon skeletons. They are widely present in plants such as *Salvia miltiorrhiza*, *C. longa*, and *Artemisia annua*. Their anti-EMs effects are mainly related to the regulation of mitochondrial autophagy and inhibition of mitochondrial division.

Ginsenosides, the characteristic triterpenoid saponins of *Panax* species, including ginseng, notoginseng, and American ginseng, comprise a structurally diverse and pharmacologically active family of phytochemicals with more than one hundred distinct aglycone-glycoside variants. They are synthesized in multiple plant tissues, including roots, leaves, stems, and fruits, and are important regulators of cellular resilience. Among them, ginsenoside Rg3 has emerged as a prominent anti-endometriosis (EMs) compound because of its broad effects on immune modulation, redox regulation, anti-angiogenesis, pro-apoptotic signaling, and mitochondrial function (Xie et al., 2024). *In vivo*, intraperitoneal administration of Rg3 in rat models of allogeneic ectopic endometrium induces marked lesion regression. It significantly reduces systemic estradiol (E_2_) and vascular endothelial growth factor (VEGF) levels, selectively inhibits the VEGF receptor-2-driven PI3K/Akt/mTOR signaling pathway within ectopic lesions, suppresses pathological neovascularization, and reactivates intrinsic apoptosis through cytochrome c release and caspase-3 cleavage, thereby disrupting the hormonal, angiogenic, and survival pathways required for EMs persistence (Liu et al., 2024). Complementing these effects, integrated *in vivo* and *in vitro* studies have shown that Rg3 also has a strong anti-fibrotic effect. It downregulates microRNA-27b-3p (miR-27b-3p), an important epigenetic regulator of TGF-β-induced fibroblast activation and extracellular matrix deposition, leading to suppression of ectopic stromal cell proliferation, inhibition of MMP-9-mediated invasion, and reduction of collagen I/III and α-SMA accumulation (Ritchie et al., 2026). At the mitochondrial level, Rg3 acts as a broad regulator of mitochondrial quality control. It enhances oxidative phosphorylation (OXPHOS) efficiency by stabilizing Complex I and ATP synthase activity, stimulates PGC-1α-NRF1-TFAM-mediated biogenesis to replenish functional mitochondria, preserves mitochondrial membrane potential (ΔΨm), inhibits mPTP opening, and modulates mitophagy through Parkin recruitment and LC3-II lipidation, thereby supporting both quantitative expansion and qualitative renewal of the mitochondrial network (Lu et al., 2025). Its antioxidant actions are also extensive. Rg3 activates the Keap1-Nrf2-ARE pathway to increase HO-1, NQO1, and SOD2 expression, suppresses NF-κB-driven inflammation, modulates Wnt/β-catenin-mediated metabolic reprogramming, and reinforces PI3K/Akt-dependent survival signaling, not as a nonselective anti-apoptotic agent, but as a context-dependent regulator that maintains mitochondrial integrity in healthy cells while allowing apoptosis in damaged or dysregulated cells (Meresman et al., 2021). In addition, Rg3 exerts specific membrane-stabilizing effects by downregulating sphingosine-1-phosphate lyase 1 (SGPL1) and the pro-apoptotic protein Bax, while upregulating the anti-apoptotic protein Bcl-2. These changes reinforce mitochondrial outer membrane integrity, prevent cytochrome c leakage, and maintain the electrochemical gradient required for bioenergetic stability (Iqbal and Rhee, 2025). Thus, ginsenoside Rg3 functions as a mitochondria-integrated therapeutic agent that coordinates hormonal regulation, vascular normalization, suppression of fibrosis, redox homeostasis, and mitochondrial renewal in endometriosis.

Tanshinone IIA (Tan II A), a lipophilic diterpenoid quinone and the principal bioactive constituent of *S. miltiorrhiza* (Danshen) roots, is a pharmacologically active compound with anti-inflammatory, antioxidant, anti-angiogenic, neuromodulatory, and mitochondria-reparative properties. More than a conventional terpenoid, Tan II A acts as a regulator of pain signaling and mitochondrial function in endometriosis (EMs). It suppresses aberrant nociceptive signaling, disrupts lesion-supportive microenvironments, and restores mitochondrial bioenergetic integrity, thereby addressing pain, disease progression, and cellular dysfunction simultaneously. In autologous EMs rat models, intraperitoneal administration of Tan II A produces marked analgesic effects. It suppresses pathological axonal sprouting in dorsal root ganglia (DRG) by downregulating estradiol (E_2_), angiotensin II (Ang II), and angiotensin II type 2 receptor (AT_2_R) expression, which are key mediators of neurogenic inflammation and peripheral sensitization, resulting in a dose-dependent increase in mechanical withdrawal threshold and clear improvement of chronic pelvic hyperalgesia (Yin et al., 2024). *In vitro*, Tan II A exerts multi-target inhibitory effects on EMs lesion viability. It suppresses TNF-α and IL-1β secretion from activated macrophages and stromal cells, downregulates matrix metalloproteinase-9 (MMP-9) and vascular endothelial growth factor (VEGF), thereby reducing extracellular matrix degradation, cell invasion, and pathological angiogenesis, and significantly decreases adhesion formation between ectopic lesions and adjacent peritoneal surfaces. Together, these effects disrupt the invasive, adhesive, and vascular processes required for lesion establishment and progression (Li et al., 2022). Notably, Tan II A also modulates the TGF-β/Smad pathway, not as a uniform fibrotic regulator, but in a context-dependent manner that promotes epithelial re-differentiation, reduces stromal hyperactivation, and suppresses expression of invasion-associated genes such as *SNAIL* and *TWIST* and apoptosis-resistance genes such as *Bcl-xL*, thereby improving tissue architecture and restoring apoptotic responsiveness in EMs models (Zhang et al., 2023). At the mitochondrial level, Tan II A acts as both a structural and functional restorer. It rescues electron transport chain (ETC) integrity by reactivating Complexes I, III, and IV, directly reversing oxidative damage to iron-sulfur clusters and heme cofactors, which leads to restored ATP synthesis, normalization of mitochondrial membrane potential (ΔΨm), and reduced ROS leakage (Li et al., 2025). It also promotes repair of the inner and outer mitochondrial membranes, stabilizes cardiolipin content, and preserves cristae density, all of which are essential for maintaining proton motive force and respiratory efficiency. In addition, Tan II A protects vascular endothelial cells from H_2_O_2_-induced oxidative injury by enhancing mitochondrial resilience. It prevents ΔΨm collapse, inhibits cytochrome c release, and upregulates mitochondrial antioxidant enzymes such as SOD2 and Trx2, thereby preserving microvascular integrity and reducing endothelial dysfunction, which is an important contributor to peritoneal hypoxia and inflammatory amplification in EMs (Ilie et al., 2026). Thus, Tan II A functions as an integrated neuro-mitochondrial therapeutic agent that simultaneously regulates pain signaling, remodels the lesion microenvironment, and restores the energetic function of diseased cells.

### 5.4 Flavonoid active components of plants

Flavonoids are among the most abundant active components in plants. They are widely distributed in plants such as honeysuckle, kudzu root, and *Scutellaria baicalensis*, and possess multiple biological activities, including anti-inflammatory, antioxidant, and anti-proliferative effects. Their therapeutic potential in EMs may be related to their ability to regulate mitochondrial quality control (MQC) and improve mitochondrial function.

Quercetin, a widely distributed flavonol and one of the most versatile dietary polyphenols, is found in capers, onions, apples, berries, and *Ginkgo biloba*. It acts as an important regulator of redox homeostasis, cellular senescence, and mitochondrial function. Its pharmacological profile is broad and includes anti-inflammatory, antioxidant, immunomodulatory, anti-angiogenic, anti-fibrotic, metabolic, and epigenetic effects, all of which contribute to interference with the major pathological features of endometriosis (EMs), including chronic inflammation, oxidative stress, and abnormal tissue remodeling. *In vivo*, sustained quercetin administration in EMs rat models induces marked lesion regression. Ectopic cysts show significant volumetric reduction, the endometrial epithelium exhibits clear atrophy and glandular simplification, and the expression of heat shock protein 70 (HSP70), a chaperone that stabilizes misfolded proteins and promotes lesion survival under stress, as well as vascular endothelial growth factor (VEGF), a key driver of pathological neovascularization, is significantly reduced in ectopic tissues (Yadav et al., 2022). *In vitro*, quercetin exerts multi-level regulatory effects on ectopic endometrial stromal cells (hESCs). It inhibits the pro-survival Akt and ERK1/2 signaling pathways, thereby reducing proliferation and resistance to apoptosis, while also enhancing p53 stability and transcriptional activity, restoring its tumor-suppressive role and promoting hormonally responsive decidualization, a physiological process whose impairment contributes to EMs implantation and persistence (Yu J. et al., 2025). In addition, quercetin suppresses invasive potential by downregulating the tight junction protein CLDN6, which paradoxically promotes motility in EMs, as well as the proliferative marker PCNA and the matrix-degrading enzymes MMP-2 and MMP-9, thereby interfering with the molecular processes required for lesion adhesion, infiltration, and expansion (Kalinová et al., 2025). At the mitochondrial level, quercetin functions as a broad regulator of redox balance and mitochondrial quality control. It maintains oxidative homeostasis not only through direct scavenging, but also through two major mechanisms: (i) enhancement of enzymatic antioxidant defenses, including SOD2, catalase, GPx, and HO-1, through activation of the Keap1-Nrf2-ARE pathway; and (ii) reinforcement of non-enzymatic antioxidant systems through regeneration of glutathione and α-tocopherol. Importantly, it also targets ROS-sensitive signaling pathways with selectivity by suppressing PI3K/Akt/mTOR hyperactivation, which drives uncontrolled growth, activating AMPK/SIRT1 to inhibit NF-κB-mediated inflammation, and modulating MAPK/AP-1 to correct stress-induced transcriptional dysregulation (Goleij et al., 2024). Beyond redox regulation, quercetin also promotes mitochondrial renewal. It stimulates PGC-1α-NRF1-TFAM-driven biogenesis, increasing mtDNA copy number and cytochrome c content; stabilizes mitochondrial membrane potential (ΔΨm); enhances Complex I-IV activity and oxidative phosphorylation (OXPHOS) efficiency; increases ATP synthesis fidelity; and activates Parkin-dependent mitophagy to eliminate damaged organelles, thereby supporting both quantitative expansion and qualitative restoration of the mitochondrial network (Sirotkin et al., 2025; Afşin et al., 2026). Thus, quercetin acts not only as a dietary flavonoid, but also as a system-level therapeutic agent that suppresses inflammation, restores hormonal responsiveness, limits invasive progression, and rebuilds the energetic and redox framework required for endometrial homeostasis and EMs resolution.

Luteolin, a bioactive flavone abundantly present in honeysuckle (*Lonicera japonica*), *Sophora flavescens*, *S. baicalensis* (Huangqin), and celery seed, is a structurally important and pharmacologically active phytochemical with effects on neuromodulation, redox regulation, inflammatory signaling, and mitochondrial dynamics. More than a conventional antioxidant, luteolin functions as a selective inducer of apoptosis and a regulator of mitochondrial homeostasis in endometriosis (EMs). It promotes caspase-dependent cell death in ectopic lesions while restoring organelle homeostasis, rebalancing metabolic activity, and reducing inflammatory recruitment, thereby suppressing lesion viability at multiple levels. *In vitro*, luteolin exerts strong pro-apoptotic effects in human EMs 12Z cells. It activates both the extrinsic apoptotic pathway through caspase-8 and the intrinsic pathway through caspase-9, ultimately resulting in cleavage of executioner caspase-3. At the same time, it suppresses expression of C-C chemokine ligand 2 (CCL2/MCP-1) and CCL5 (RANTES), which are important chemoattractants for monocytes and macrophages, thereby disrupting the inflammatory microenvironment that supports lesion survival, angiogenesis, and fibrosis (Tesarik et al., 2025). *In vivo*, intraperitoneal administration of luteolin in autologous EMs mouse models causes clear lesion regression, with significant volumetric reduction and structural simplification of ectopic implants. Mechanistically, luteolin imposes strong cell cycle regulation by downregulating cyclin E1 (CCNE1) and its catalytic partners CDK2 and CDK4, thereby inducing G1/S phase arrest and preventing DNA replication licensing. At the same time, it inhibits the PI3K/Akt/MAPK oncogenic pathway and suppresses CCNE1 transcriptional activation, thereby removing proliferative autonomy in ectopic stromal cells (Raslan et al., 2026). In addition, luteolin targets the prostaglandin E_2_ (PGE_2_) pathway, which is a central contributor to EMs-associated pain and disease progression, by upregulating 15-hydroxyprostaglandin dehydrogenase (HPGD), the rate-limiting enzyme responsible for catabolizing PGE_2_ into inactive metabolites. This reduces EP2/EP4 receptor signaling, downregulates COX-2 and MMP-9 expression, and suppresses epithelial-mesenchymal transition (EMT), collectively reducing stromal cell invasion, migration, and proliferative expansion (Woo et al., 2021). At the mitochondrial level, luteolin functions as a regulator of mitochondrial quality control through active remodeling rather than static protection. It promotes balanced mitochondrial fission through modulation of Drp1 phosphorylation and fusion through upregulation of MFN1/2 and OPA1, enhances Parkin-mediated mitophagy to clear depolarized organelles, and activates PGC-1α-NRF1-TFAM-driven biogenesis to replenish functional mitochondria (Tian et al., 2025). It also improves bioenergetic function by increasing the activity of oxidative phosphorylation (OXPHOS) complexes I-V, elevating mitochondrial membrane potential (ΔΨm), enhancing ATP synthesis efficiency, and reducing electron leakage, thereby shifting mitochondria from sources of pathological ROS toward effective energy-producing organelles (Zhang et al., 2026). Thus, luteolin functions not only as a flavonoid, but also as an integrated therapeutic agent that promotes targeted apoptosis, arrests cell cycle progression, suppresses inflammatory chemokine networks, reduces PGE_2_-driven pathology, and restores mitochondrial structure and function to improve endometrial homeostasis in EMs.

### 5.5 Polysaccharide-based active components of plants

Plant polysaccharides are high-molecular-weight compounds widely distributed in the roots, stems, and leaves of various natural plants. They have multiple medicinal properties, including immune regulation, antioxidant activity, anti-tumor effects, and antiviral and antibacterial activities. With increasing investigation into their mechanisms of action, numerous studies have confirmed that plant polysaccharides have significant therapeutic effects on EMs.

Spirulina polysaccharide, a bioactive heteropolysaccharide complex isolated from the cyanobacterium *Arthrospira platensis*, functions as a potent multi-target immunometabolic modulator in endometriosis (EMs). It acts as more than a simple anti-inflammatory agent, exerting coordinated suppression of lesion growth, angiogenesis, extracellular matrix remodeling, and pro-survival signaling, thereby inhibiting disease progression at multiple molecular levels. *In vivo*, oral or intraperitoneal administration of spirulina polysaccharide in EMs rat models produces marked therapeutic effects. Ectopic lesions show significant volumetric regression, while systemic and local production of key pathological mediators, including tumor necrosis factor-alpha (TNF-α), vascular endothelial growth factor (VEGF), and matrix metalloproteinase-2 (MMP-2), is markedly reduced. In addition, the hyperactivated PI3K/Akt/mTOR signaling pathway is strongly inhibited, collectively disrupting the inflammatory, vascular, and proliferative mechanisms that support EMs persistence (Shi FL. et al., 2026). At the mitochondrial level, spirulina polysaccharide exerts substantial restorative effects, particularly in metabolically impaired cells. *In vitro* studies using aged human dermal fibroblasts, a validated model of mitochondrial aging, show that the spirulina polysaccharide complex (SPC) markedly upregulates the mitochondrial antioxidant enzyme superoxide dismutase 2 (SOD2), increasing both its transcriptional expression and enzymatic activity. This induction of SOD2 effectively scavenges mitochondrial superoxide (O_2_•^-^), prevents oxidative injury to electron transport chain (ETC) complexes and mtDNA, restores mitochondrial membrane potential (ΔΨm), improves respiratory control ratio, and reactivates collagen biosynthesis, which is a key functional process impaired by mitochondrial dysfunction in stromal fibroblasts within EMs lesions (Brasil et al., 2023). Thus, spirulina polysaccharide acts as a dual-function therapeutic by suppressing pathological signaling pathways while simultaneously restoring mitochondrial energetic and redox homeostasis, which are fundamental to cellular repair, tissue homeostasis, and EMs resolution.

Laminarin, a β-1,3-glucan polysaccharide enriched in brown algae such as *Laminaria japonica* (kombu) and *Ascophyllum nodosum*, is a marine-derived immunomodulator whose therapeutic action in endometriosis (EMs) is mediated through selective suppression of stress-activated kinase signaling and pathological angiogenesis. In EMs rat models, laminarin administration significantly reduces aberrant activation of the mitogen-activated protein kinase (MAPK) pathway within large ectopic endometrial lesions. Specifically, it downregulates phosphorylated p38 MAPK (p-p38) relative to total p38 and phosphorylated ERK1/2 (p-ERK1/2) relative to total ERK1/2, thereby suppressing two major stress-responsive pathways involved in inflammation, cell proliferation, and survival in hypoxic and oxidative microenvironments. This modulation of kinase signaling results in clear functional effects. Laminarin strongly inhibits VEGF-driven neovascularization in ectopic tissues, reduces endothelial tube formation, and induces volumetric regression of established lesions, thereby limiting EMs progression at both molecular and anatomical levels (Ramanathan et al., 2025). At the mitochondrial level, laminarin acts as a regulator of organelle homeostasis in metabolically stressed cells. *In vitro* studies show that kelp-derived polysaccharides, including laminarin, reduce calcium dyshomeostasis by preventing pathological mitochondrial Ca^2+^ overload, which is a major trigger of mPTP opening, cytochrome c release, and intrinsic apoptosis. Importantly, this regulation is context-dependent. In abnormally proliferating ectopic endometrial cells, laminarin permits ROS-mediated apoptosis and endoplasmic reticulum (ER) stress, not as uncontrolled toxicity, but as a regulated mechanism for the elimination of dysfunctional cell populations. It achieves this by modulating mitochondrial-ER cross-talk, reducing Ca^2+^ flux across mitochondria-associated membranes (MAMs), decreasing CHOP and GRP78 expression, which are markers of unresolved ER stress, and allowing selective activation of caspase-12 and JNK. In this way, laminarin links redox imbalance to controlled apoptotic execution without inducing necrotic inflammation (Machihara et al., 2023). Thus, laminarin functions as a stress-responsive therapeutic agent that modulates kinase signaling, stabilizes mitochondrial-ER communication, and directs diseased cells toward regulated resolution rather than persistent survival.

Seaweed polysaccharide, a structurally diverse group of sulfated and non-sulfated glycans derived from brown algae such as *Fucus vesiculosus* and *Undaria pinnatifida* and red algae such as *Porphyra umbilicalis*, acts as a systemic endocrine-immune-mitochondrial modulator in endometriosis (EMs). Its therapeutic effects do not arise from a single biochemical action, but from integrated regulation of hormonal signaling, inflammatory status, and mitochondrial bioenergetics. In EMs rat models, seaweed polysaccharide administration produces marked endocrine normalization. It significantly reduces serum levels of follicle-stimulating hormone (FSH) and luteinizing hormone (LH), which are key gonadotropins that promote estrogen-dependent lesion growth, while also lowering pro-inflammatory interleukin-6 (IL-6) and the lipid peroxidation marker malondialdehyde (MDA). This combined suppression of endocrine drivers and oxidative-inflammatory mediators helps restore hypothalamic-pituitary-ovarian axis homeostasis, reduce chronic pelvic inflammation, and improve systemic antioxidant capacity, ultimately leading to measurable reductions in lesion burden, glandular hyperplasia, and stromal fibrosis (Polat and Ozogul, 2026). At the mitochondrial level, seaweed polysaccharide acts as a broad structural and functional regulator. It preserves ETC integrity by protecting complexes I, III, and IV against oxidative inactivation; maintains mitochondrial membrane architecture by stabilizing cardiolipin content and preventing phospholipid peroxidation; preserves mitochondrial DNA (mtDNA) copy number and integrity against ROS-induced strand damage; regulates Ca^2+^ flux across mitochondrial membranes to prevent pathological overload and mPTP opening; activates Parkin-PINK1-mediated mitophagy to remove depolarized organelles; and balances mitochondrial fission through Drp1 modulation and fusion through OPA1 and MFN2 upregulation to maintain a dynamic and functional mitochondrial network (Sato et al., 2025). These actions are interrelated and mutually reinforcing. Improved ETC efficiency reduces ROS leakage, which helps protect mtDNA and mitochondrial membranes. Stabilized Ca^2+^ homeostasis prevents cristae remodeling and cytochrome c release, while balanced mitochondrial dynamics support optimal distribution of functional mitochondria to regions with high energy demand. Thus, seaweed polysaccharide functions as a broad mitochondrial regulator that simultaneously reduces endocrine dysregulation, resolves inflammatory and oxidative stress, and restores the energetic framework required for endometrial tissue repair and EMs resolution.

### 5.6 Naphthoquinone plant active components

It belongs to the quinone class of compounds. Most naturally occurring members are α-naphthoquinone derivatives, typically appearing as orange or orange-red crystals, with a smaller proportion presenting as purple.

Shikonin, a red naphthoquinone pigment and major bioactive constituent of the roots of *P. cuspidatum* (Japanese knotweed, Hu Zhang), is a pharmacologically active compound with effects on immune regulation, endocrine function, chemokine signaling, and cellular metabolism. In endometriosis (EMs), it acts across the peritoneal immune, endocrine, and stromal environment. It reduces inflammatory cell recruitment, contributes to hormonal regulation, interferes with chemokine-mediated lesion establishment, and modulates mitochondrial biogenesis in a context-dependent manner.

*In vivo*, administration of shikonin in severely immunodeficient murine EMs models significantly reduces monocyte infiltration into the peritoneal cavity. This effect is associated with downregulation of RANTES (CCL5), a T-cell–derived chemokine involved in macrophage polarization and lesion-associated angiogenesis. As a result, peritoneal inflammation is reduced, fibrotic adhesions surrounding ectopic implants are decreased, and lesion expansion is inhibited (Wang Y. et al., 2026).

In addition to its immunomodulatory effects, shikonin influences endocrine regulation in EMs rat models. It reduces elevated estradiol (E_2_) levels while increasing serum follicle-stimulating hormone (FSH), luteinizing hormone (LH), and progesterone, contributing to restoration of the hypothalamic–pituitary–ovarian axis and hormonal balance. It also disrupts the SDF-1/CXCR4 chemotactic axis by downregulating stromal-derived factor-1 (SDF-1/CXCL12) and its receptor CXCR4. This pathway is involved in progenitor cell recruitment, angiogenesis, and stromal cell survival. Inhibition of this signaling axis results in reduced lesion volume and limits disease progression (Park et al., 2023).

At the mitochondrial level, shikonin regulates mitochondrial biogenesis through pathway-specific mechanisms. Rather than broadly reducing mitochondrial content, it modulates the PGC-1α signaling axis. Shikonin increases expression of glycogen synthase kinase-3β (GSK-3β) and promotes its interaction with PGC-1α. This interaction facilitates phosphorylation of PGC-1α at Thr295, which reduces its transcriptional coactivator activity. As a result, downstream targets of the PGC-1α/ERRα pathway, including NRF1, TFAM, and mitochondrial-encoded electron transport chain subunits, show reduced expression, leading to controlled suppression of mitochondrial biogenesis (Wang H. et al., 2026).

This regulation is relevant in EMs, where increased mitochondrial biogenesis contributes to oxidative stress, altered metabolic activity, and lesion growth. Through these combined effects on immune signaling, endocrine regulation, chemokine pathways, and mitochondrial function, shikonin demonstrates a multi-level mechanism of action in EMs.

## 6 Current issues in the research

Although substantial progress has been made in recent years in understanding how botanical therapeutics regulate mitochondrial quality control (MQC) in endometriosis (EMs), important translational barriers remain. Studies have identified molecular targets of phytoactive compounds and clarified several regulatory mechanisms involved in their effects on EMs. However, major challenges continue to limit clinical translation. These include pharmacokinetic instability related to poor oral bioavailability and rapid metabolism, insufficient tissue-specific delivery to pelvic lesions, limited mechanistic validation in human-relevant 3D organoid and patient-derived xenograft models, a lack of standardized and clinically graded botanical preparations, and a shortage of large-scale randomized controlled trials evaluating efficacy, safety, and long-term outcomes across diverse patient populations. Together, these limitations hinder the translation of phytomedicine from experimental findings to routine clinical application.

First, the translational pathway remains limited. Although important findings have been generated from *in vitro* cell culture studies and rodent EMs models, clinical investigation remains relatively underdeveloped. Human studies are few and are largely restricted to small pilot trials with short follow-up periods, limited statistical power, and non-standardized outcome measures. In addition, optimal treatment regimens, including dosage, route of administration such as oral, intraperitoneal, or nanoparticle-based delivery, and treatment duration, remain poorly defined for most phytoactive compounds. This lack of evidence makes it difficult to draw clear conclusions regarding clinical efficacy and safety. The problem is further complicated by marked interpatient heterogeneity. EMs varies widely in lesion distribution, inflammatory burden, hormonal responsiveness, age-related ovarian reserve, and reproductive priorities. However, personalized phytomedicine strategies tailored to disease endotype, metabolic phenotype, and treatment goals remain largely theoretical, which limits treatment consistency, reproducibility, and real-world effectiveness.

Second, mechanistic understanding remains incomplete. Current research has focused mainly on isolated modulation of individual MQC components, such as mitophagy, biogenesis, or fission, while giving relatively little attention to the coordinated and interdependent interactions among mitochondrial dynamics, mitophagy, biogenesis, and proteostasis. The upstream regulatory network is also not fully defined. It remains unclear how phytochemicals interact with master kinases such as AMPK, PKA, and GSK-3β, nutrient sensors such as SIRT1 and mTOR, and redox-sensitive transcription factors such as Nrf2, PGC-1α, and FOXO3. Similarly, downstream effects, including changes in metabolic reprogramming between oxidative phosphorylation and glycolysis, ROS signaling, and calcium buffering capacity, are rarely examined in an integrated manner. In particular, mitochondrial protein homeostasis, including chaperone-assisted folding through HSP60 and HSP10, proteolytic degradation through LONP1 and CLPP, and ribosomal surveillance, remains an underexplored area in phytomedicine research. At present, there are no published studies directly examining how botanical compounds regulate this important component of MQC.

Third, pharmaceutical limitations continue to restrict therapeutic potential. Many key phytoactive compounds, including curcumin, resveratrol, epigallocatechin gallate (EGCG), and quercetin, have unfavorable pharmacokinetic properties, including poor aqueous solubility, extensive phase II metabolism through glucuronidation and sulfation, rapid systemic clearance, and limited membrane permeability. These properties result in low tissue concentrations, particularly within pelvic lesions protected by peritoneal barriers. At the same time, analytical standardization remains inconsistent. Variation in active compound content between batches, influenced by plant genetics, harvest season, geographic origin, drying conditions, and extraction methods, reduces experimental reproducibility and weakens confidence in preclinical findings. Without certified reference materials, validated HPLC-MS quantification, and Good Manufacturing Practice (GMP)-compliant production, phytomedicine is unlikely to progress into evidence-based clinical use.

Fourth, disciplinary separation continues to limit innovation. The development of mitochondria-targeted phytomedicine requires close integration across plant chemistry, systems pharmacology, live-cell imaging, mitochondrial metabolomics, 3D organoid pathophysiology, and clinical trial methodology. However, collaboration among botanists, mitochondrial biochemists, computational modelers, and gynecologic clinicians remains limited. This fragmentation slows hypothesis generation, delays mechanistic validation, and weakens the iterative bench-to-bedside process needed to translate molecular findings into meaningful clinical outcomes.

Beyond its canonical roles in bioenergetics and quality control, mitochondrial function is increasingly recognized as a key regulator of epigenetic remodeling and cell fate. Mitochondrial metabolism directly influences the availability of acetyl-CoA, a central substrate for histone acetylation. Foundational studies have demonstrated that ATP-citrate lyase links mitochondrial-derived citrate metabolism to nuclear histone acetylation, establishing a direct connection between cellular metabolism and chromatin regulation (Wang et al., 2024). Furthermore, emerging evidence indicates that mitochondrial pathways such as fatty acid oxidation and mitochondrial dynamics, including fusion and cristae remodeling, can modulate intracellular acetyl-CoA pools and histone acetylation status, thereby influencing gene expression programs and cell differentiation (Wellen et al., 2009; Wong et al., 2017; Wu et al., 2022). These findings suggest that mitochondrial quality control (MQC) extends beyond energy homeostasis to actively shape epigenetic states and cellular identity. In the context of endometriosis, where aberrant proliferation, survival, and differentiation are central features, MQC-driven metabolic reprogramming may contribute to disease progression through metabolite-dependent epigenetic regulation.

## 7 Future research directions

In light of the critical gaps identified in current research—and building upon the mechanistic foundations already established—the future trajectory of phytomedicine for endometriosis (EMs) must move toward clinical translation, deeper systems-level understanding, pharmaceutical standardization, interdisciplinary integration, and rigorous safety evaluation. Five strategic priorities define the path forward:

First, accelerate clinical development through well-designed, patient-centered trials. There is a need to move beyond pilot-scale studies to multicenter, randomized, double-blind, placebo-controlled trials with adequately powered sample sizes, extended follow-up (≥24 months), and standardized, clinically validated endpoints, including pain assessment (VAS/NRS), fertility outcomes (time-to-pregnancy, live birth rates), lesion volume quantification using MRI, and quality-of-life measures such as ENDO-QoL. Dose-finding studies are essential to determine not only efficacy but also therapeutic windows, including optimal dosage, route of administration (e.g., oral nanoformulations or intraperitoneal sustained-release systems), and treatment duration for each compound. At the same time, precision approaches should be incorporated by developing stratified treatment protocols based on EMs subtypes (peritoneal, ovarian, deep infiltrating), hormonal profiles (e.g., estrogen-dominant or progesterone-resistant), and reproductive goals such as fertility preservation or symptom management. In addition, the potential for combination therapy should be systematically evaluated, including integration with first-line hormonal treatments such as GnRH analogues and progestins, as well as post-surgical adjuvant use, with the aim of reducing side effects, preventing recurrence, and prolonging remission. Biomarker-guided monitoring, including changes in CA125, HE4, VEGF, and microRNA profiles, may further support treatment evaluation and real-time adjustment.

Second, mitochondrial quality control (MQC) should be investigated as an integrated and adaptive network rather than as isolated pathways. Future work should focus on the four interconnected components of MQC: mitochondrial dynamics (Drp1/MFN/OPA1), mitophagy (PINK1/Parkin/LC3-II), biogenesis (PGC-1α/NRF1/TFAM), and proteostasis (HSP60/HSP10, LONP1, CLPP). It is also important to examine upstream regulatory pathways, including nutrient-sensing pathways (AMPK, SIRT1), redox regulators (Nrf2, FOXO3), and signaling kinases (GSK-3β, Akt), and to determine how phytochemicals interact with these systems. Downstream consequences should also be characterized, including effects on metabolic flexibility (balance between oxidative phosphorylation and glycolysis), ROS signaling regulation, calcium buffering capacity, and mtDNA integrity. In particular, mitochondrial proteostasis remains insufficiently studied and requires focused investigation, including identification of plant-derived compounds that regulate mitochondrial chaperones and proteases.

Third, development should focus on pharmaceutical-grade phytotherapeutics rather than unstandardized botanical extracts. Pharmacokinetic limitations should be addressed through advanced formulation strategies, including lipid-polymer hybrid nanoparticles, cyclodextrin inclusion complexes, and PEGylated micelles, to improve solubility, bioavailability, circulation time, and tissue targeting. Delivery systems should also be designed to respond to the specific microenvironment of endometriotic lesions. For example, hypoxia-responsive systems may enable localized drug release and improved retention within the pelvic cavity, thereby increasing therapeutic precision while reducing systemic exposure (Liao et al., 2023; Fan et al., 2024; Friend, 2017; Li et al., 2021). At the same time, strict standardization is required, including controlled cultivation practices (GAP), defined harvesting protocols, standardized extraction methods (e.g., supercritical CO_2_ or ethanol-based extraction), purification techniques such as preparative HPLC, and formulation under Good Manufacturing Practice (GMP) conditions. Comprehensive quality control measures, including reference standards, HPLC-HRMS fingerprinting, contaminant testing (heavy metals and residual solvents), and microbiological assessment, are necessary to ensure reproducibility and regulatory compliance.

Fourth, interdisciplinary collaboration should be strengthened. Progress in this field requires integration across plant chemistry, mitochondrial biology, gynecology, and systems biology. This includes collaboration between experimental and clinical researchers, as well as integration of traditional medical approaches, such as pattern differentiation in Traditional Chinese Medicine, with modern multi-omics methods including metabolomics and single-cell RNA sequencing. Computational approaches, including AI-based network pharmacology, may help identify multi-target interactions and guide experimental validation in advanced models such as organoids and patient-derived xenografts. This combined approach may support the development of more precise, mechanism-based therapies using plant-derived compounds.

Fifth, safety assessment should be incorporated as a core component of development. This includes comprehensive toxicological evaluation, such as subchronic and chronic toxicity studies, genotoxicity testing (e.g., Ames and micronucleus assays), reproductive toxicity studies, and drug–drug interaction assessments, particularly for compounds affecting CYP450 pathways. Safe dosage ranges should be defined using NOAEL and LOAEL approaches. In addition, post-marketing surveillance systems should be established to monitor long-term safety. Ensuring safety throughout all stages of development, from preclinical studies to clinical application, is essential for the responsible use of phytomedicine.

## 8 Limitations

Despite the promising therapeutic potential of phytochemicals in endometriosis, several limitations should be considered. Many plant-derived compounds exhibit low oral bioavailability due to poor solubility, rapid metabolism, and limited absorption, which may reduce their therapeutic efficacy *in vivo*. In addition, variability in plant sources, extraction methods, and compound purity can lead to challenges in standardization and reproducibility across studies. Furthermore, most current evidence is derived from *in vitro* experiments or animal models, and well-designed clinical trials evaluating the safety, optimal dosing, and long-term efficacy of these compounds in endometriosis patients remain limited. Future research should therefore focus on improving delivery systems, standardizing phytochemical preparations, and conducting rigorous clinical investigations to facilitate translation into clinical practice.

## 9 Conclusion

Endometriosis (EMs), a pervasive, estrogen-driven, inflammatory gynecologic disorder affecting up to 10% of reproductive-aged women, is far more than a benign anatomical anomaly. It is a systemic disease that impairs fertility, intensifies chronic pelvic pain, disrupts hormonal balance, and substantially reduces physical, emotional, and social wellbeing. Current therapeutic approaches, including hormonal suppression, surgical excision, and empirical analgesia, remain limited by high recurrence rates, ovarian toxicity, surgical morbidity, and symptomatic relapse, highlighting an urgent unmet need for safe, sustainable, and mechanism-based interventions. At the center of EMs pathogenesis lies marked dysregulation of mitochondrial quality control (MQC). Defective fission-fusion dynamics, impaired mitophagic clearance, suppressed biogenesis, and disrupted proteostasis converge to produce bioenergetic failure, pathological ROS overproduction, calcium dyshomeostasis, and resistance to apoptosis, thereby transforming mitochondria from cellular powerhouses into drivers of lesion persistence and inflammation.

Phytomedicine emerges as a particularly promising therapeutic paradigm. Its inherent polypharmacology allows simultaneous modulation of multiple MQC components, its favorable safety profile supports long-term use, and its broad structural diversity, ranging from flavonoids such as quercetin and luteolin, to terpenoids such as tanshinone IIA and shikonin, to polyphenols such as resveratrol and curcumin, provides a rich source of mitochondria-targeting molecules. Increasing preclinical evidence shows that these phytoactive compounds restore mitochondrial integrity in ectopic endometrial cells. They reactivate PGC-1α-driven biogenesis, rebalance Drp1/MFN-mediated dynamics, enhance Parkin-PINK1-dependent mitophagy, promote superoxide scavenging through SOD2 upregulation, suppress NLRP3 inflammasome activation, inhibit abnormal proliferation, and reactivate intrinsic apoptotic pathways. Together, these effects contribute to inhibition of lesion growth, resolution of peritoneal inflammation, and restoration of tissue homeostasis. However, this therapeutic promise remains largely preclinical. Important translational barriers persist, including limited clinical validation, fragmented mechanistic understanding across isolated pathways, and pharmaceutical limitations such as poor solubility, rapid metabolism, and batch variability that reduce reproducibility. In addition, disciplinary separation continues to hinder systems-level progress. The path forward requires strategic integration: accelerating rigorous human trials, defining MQC as an integrated and adaptive network rather than a collection of isolated targets, developing pharmaceutical-grade botanical formulations, strengthening transdisciplinary collaboration across plant chemistry, mitochondrial bioenergetics, and clinical gynecology, and establishing safety as a central and non-negotiable priority. By advancing along this integrated path, phytomedicine may move beyond its traditional role and become a cornerstone of precision, mitochondria-targeted therapy for EMs, offering not only symptom relief but also meaningful disease modification and sustained restoration of reproductive health.
